# Factor H polymorphism elevates granzyme B and impairs complement inhibition in human and mouse models

**DOI:** 10.1016/j.isci.2026.117957

**Published:** 2026-10-19

**Authors:** Harshini Chakravarthy, Jeanne Xi, Hyung-Suk Yoo, Andrew Buss, Boaz Li, Robert Bell, Anne Haegert, Stephane Le Bihan, David J. Granville, Joanne A. Matsubara

**Affiliations:** 1 Department of Ophthalmology and Visual Sciences, UBC, Vancouver, BC, Canada; 2 Vancouver Prostate Centre, Vancouver Coastal Health Research Institute, Vancouver, BC, Canada; 3 International Collaboration on Repair Discoveries (ICORD), Vancouver Coastal Health Research Institute, Vancouver, BC, Canada; 4 Department of Pathology and Laboratory Medicine, UBC, Vancouver, BC, Canada

**Keywords:** cervical cancer, ELAVL1, ACVRL1, CASP14, targeted therapy

## Abstract

The complement factor H (CFH) Y402H polymorphism is a leading genetic risk factor for age-related macular degeneration (AMD). Here, we identify extracellular granzyme B (GzmB) as a crucial mediator connecting complement dysregulation to structural pathology. In non-AMD human donor eyes, the high-risk CFH genotype was associated with increased GzmB-positive choroidal mast cells, membrane attack complex deposition, and distinct vitronectin redistribution. Using *in vitro* human retinal pigment epithelium (RPE) cells, we demonstrate that complement fragment C5a triggers GzmB secretion and zonula occludens-1 disruption. RNA sequencing of GzmB-stimulated RPE cells confirmed transcriptomic signatures of barrier dysfunction and cellular stress. Furthermore, GzmB-deficient mice displayed preserved choroidal vitronectin compared with wild-type controls, supporting a role for GzmB in cleaving this terminal complement inhibitor. These findings delineate a pathogenic C5a-GzmB pathway compromising extracellular matrix integrity, implicating extracellular GzmB as a critical downstream mediator linking genetic risk to early AMD onset.

## Highlights

• High-risk CFH eyes exhibit choroidal vitronectin loss and mast cell accumulation
• Complement C5a triggers granzyme B secretion and RPE tight junction disruption
• Extracellular granzyme B links genetic risk to structural pathology in AMD

## Graphical abstract

**Figure:**
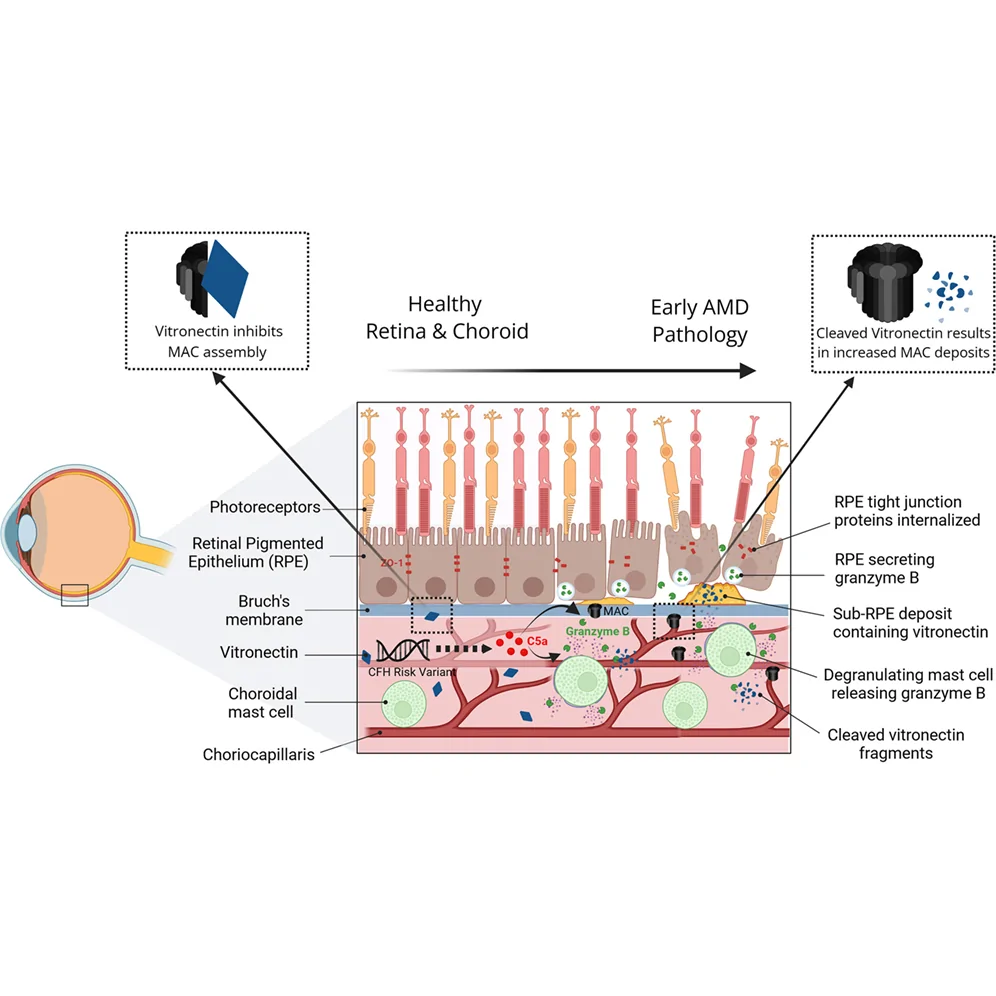

## Teaser

Biological sciences; Cell biology

## Introduction

Age-related macular degeneration (AMD) is a leading cause of irreversible vision loss.^1^ Its multifactorial pathogenesis is driven by genetics, environment, and aging.^2^^,^^3^ Despite the efficacy of anti-VEGF therapies for the neovascular (wet) form of AMD, treatment for the more prevalent dry form remains an unmet clinical need, particularly in the early stages where therapeutic intervention could prevent progression to geographic atrophy.^4^^,^^5^

Gene-association studies implicate the alternative complement system in the pathogenesis of AMD, specifically the *Y402H* variant in the complement factor H (*CFH*) gene.^6^^,^^7^ Individuals homozygous for the high-risk CC genotype exhibit a 5- to 7-fold increased risk versus the protective TT genotype.^8^ *CFH* normally acts as a negative regulator of the alternative complement pathway, preventing excessive activation.^9^ However, the *Y402H* polymorphism, located in the ligand-binding region, hinders anchoring to polyanionic ligands like heparan sulfate and C-reactive protein (CRP) on Bruch’s membrane, thereby impairing regulatory function.^10^^,^^11^

A critical gap remains in linking this genetic susceptibility to retinal structural damage. While loss of regulation explains chronic inflammation and production of small cleavage fragments or anaphylatoxins (C3a and C5a),^12^^,^^13^^,^^14^ these receptor-acting inflammatory mediators^15^^,^^16^ lack the intrinsic catalytic protease activity to explain extracellular matrix (ECM) remodeling and retinal pigmented epithelial (RPE) dysfunction characterizing early AMD.^17^^,^^18^ They cannot generate specific structural protein cleavage or calcified deposit formation.^19^^,^^20^ We therefore need to identify molecules downstream of the *CFH* polymorphism that trigger these events.

We propose a hypothesis centered on granzyme B (GzmB), a serine protease traditionally associated with cytotoxic cells,^21^ but increasingly recognized for perforin-independent, extracellular roles in ECM cleavage and epithelial dysfunction in aging and disease.^22^^,^^23^ We previously demonstrated that GzmB levels increase with age and that this protease is secreted by two primary sources: stressed RPE at Bruch’s membrane and resident mast cells within the choroid.^24^^,^^25^ We hypothesize that in non-AMD eyes with the high-risk *CFH* genotype, elevated C5a stimulates extracellular GzmB secretion.

Studies have established that GzmB targets and cleaves vitronectin,^26^^,^^27^ a multifunctional glycoprotein abundant in the choroid and in drusen.^20^^,^^28^ Vitronectin is a complement inhibitor, binding to C5b-7 and preventing insertion of membrane attack complex (MAC) into host cell membranes.^29^^,^^30^ GzmB-mediated cleavage of vitronectin would therefore result in failure of this protective function, rendering the RPE vulnerable to complement-mediated lysis. Consistent with this, we observe vitronectin in high-risk eyes aggregating in sub-RPE deposits. By examining non-AMD eyes stratified by *CFH* genotype, we characterize early pathogenic processes involving GzmB-mediated vitronectin cleavage that likely contribute to the onset of dry AMD.

## Results

### Systemic GzmB levels in dry AMD patients by CFH genotype

To determine if the localized, genotype-driven pathology of AMD is reflected in systemic biomarkers, we analyzed plasma samples from cohort 1 participants, stratifying the dry AMD patients by their *CFH* genotype (low-risk TT Vs. high-risk CC). A cohort of non-AMD, younger controls was included to establish a physiological baseline. While systemic GzmB levels in both dry AMD groups were visibly higher than the baseline (mean [SEM], 22.3 [7.5] pg/mL), comparison between the two age-matched AMD cohorts revealed no significant difference in systemic GzmB concentrations (mean [SEM], 89.49 [23.65] vs. 64.48 [24.35] pg/mL; Figure 1A). This suggests that while generalized systemic inflammatory dysregulation is a feature of dry AMD, the specific pathogenic differences driven by the *CFH Y402H* polymorphism likely occur early and locally within the retinal tissue microenvironment.

**Figure 1:**
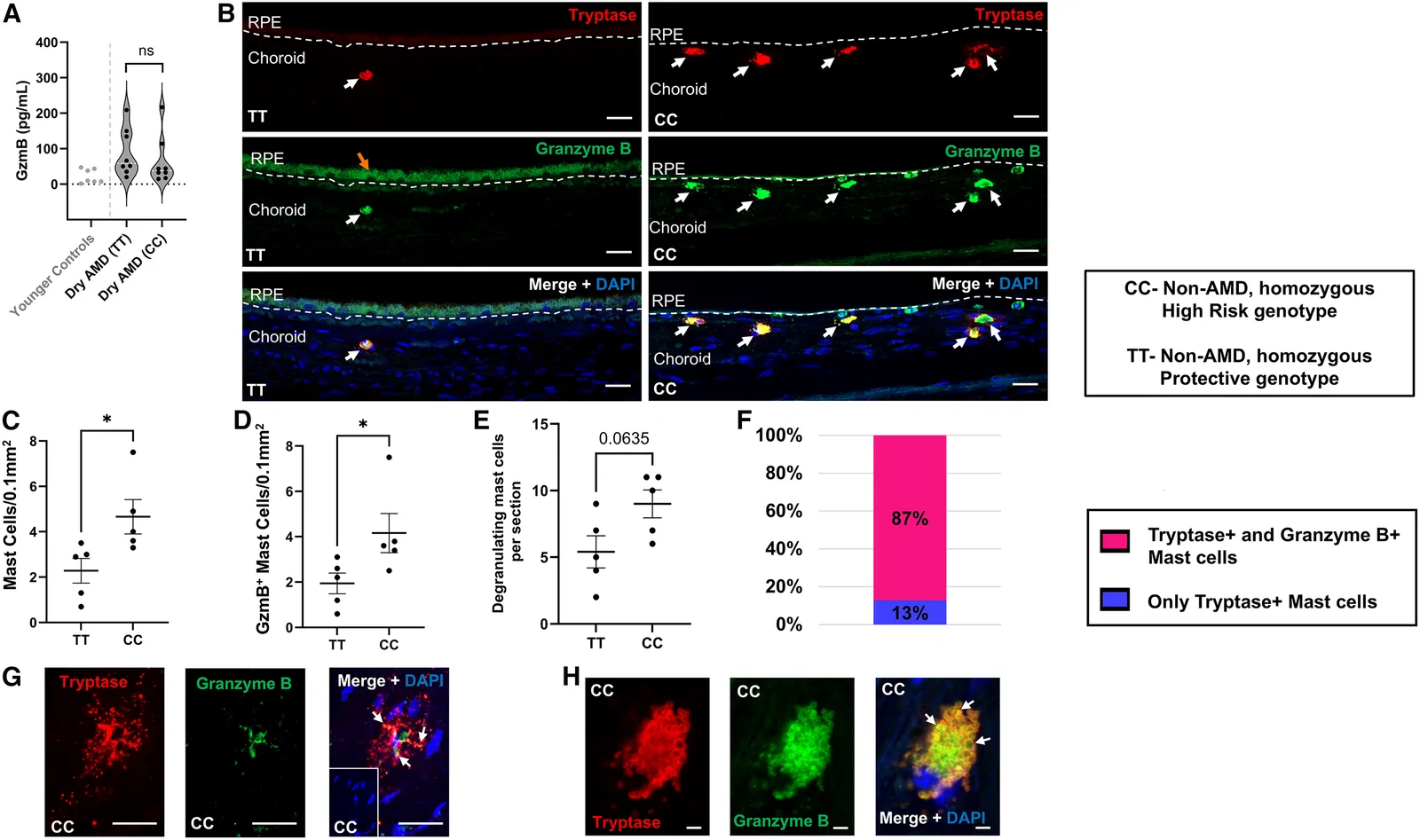
Systemic granzyme B profiles in dry AMD and localized, genotype-driven choroidal mast cell infiltration in high-risk donor eyes (A) Violin plot of systemic plasma granzyme B (GzmB) concentrations in dry AMD patients, stratified by *CFH* genotype (low-risk TT, *n*=8 patients vs. high-risk CC, *n*=8 patients). Black dots represent individual AMD patient data points used for statistical analysis. A cohort of younger, non-AMD controls (*n*=7 individuals) is displayed as gray dots to provide a physiological baseline. The dashed white line indicates the median for each group, and the black dotted line represents zero. ns, not significant (Mann-Whitney U test comparing TT and CC AMD groups; gray dashed line separates control group excluded from statistical comparison). (B) Representative immunofluorescence of protective TT (left column) and high-risk homozygous CC (right column) donor eyes (*n*=5 independent eyes per genotype). Rows display tryptase (red; top), GzmB (green; middle), and merged channels with DAPI (bottom). A white dashed line marks Bruch’s membrane, separating RPE from choroid. Note increased accumulation of GzmB+ mast cells in the CC choroid (white arrows) compared with the TT control. An orange arrow indicates variable RPE autofluorescence in green channel. Scale bars: 20 μm. (C–E) Quantification of total mast cell density (C) and GzmB+ mast cell density (D) confirms a significant increase in CC eyes (*p* =0.016 and *p*=0.032, respectively), while degranulating mast cell density (E) shows no significant difference (*p* = 0.0635). (F) Quantification showing the proportion of choroidal mast cells co-expressing GzmB and tryptase. (G) Representative image of a degranulating mast cell in the choroid of a CC donor. Scale bars: 20 μm. (H) High-magnification image demonstrating partial colocalization of tryptase (red) and GzmB (green) granules within choroidal mast cells (white arrows). Scale bars, 2 μm. In (C)–(F), individual data points for each donor eye are displayed, with horizontal lines indicating the mean ± SEM. Statistical significance for (C)–(E) was determined using Mann-Whitney U test.

### Choroidal infiltration of GzmB+ mast cells in non-AMD, genetically at-risk eyes

Recent proteomic and histological analysis identified choroidal mast cell accumulation and the release of mast cell-specific proteases such as tryptase and chymase, as early events in AMD pathogenesis driven by genetic risk at the *CFH* locus.^31^ To validate these findings and investigate the involvement of GzmB in AMD pathogenesis, we performed immunofluorescence staining on non-AMD human donor eyes genotyped for the *CFH* Y402H polymorphism. Tryptase was used as a mast cell marker. Consistent with previous findings*,*^31^ we observed significantly increased infiltration of total mast cells and GzmB+ mast cells in the choroid of non-AMD donor eyes carrying the high-risk homozygous CC variant, compared with age-matched donor eyes with the protective homozygous TT genotype (white arrows, Figures 1B–1D). Quantification showed a mean [SEM] density of 4.2 [0.8] GzmB+ mast cells/0.1 mm^2^ in the CC group compared with 1.9 [0.4] cells/0.1 mm^2^ in the TT group (Figure 1D; *p* < 0.05). Although the quantitative increase in degranulating cells in the CC cohort only trended toward significance (*p* = 0.0635; Figure 1E), the presence of extracellular GzmB+ granules supports the hypothesis that mast cell degranulation drives ECM remodeling in the early stages of AMD. While a subset of GzmB+ cells (24.2% combined average) were tryptase-negative, indicating a basal presence of other immune effectors such as natural killer or cytotoxic T cells in the choroid,^32^^,^^33^ dual-labeling demonstrated that the vast majority (87%) of resident choroidal mast cells actively co-express GzmB and tryptase (Figure 1F). This establishes mast cells as the primary endogenous immune source of GzmB in this microenvironment.

High-magnification imaging demonstrated active mast cell degranulation in a CC donor eye, evidenced by partially colocalized GzmB^+^ and tryptase^+^ granules within the surrounding ECM (Figure 1G). Finally, super-resolution microscopy revealed partial co-localization of granules containing tryptase (red) and GzmB (green) in these cells (Figure 1H).

### Loss of choroidal vitronectin in non-AMD, genetically at-risk eyes

Next, we investigated potential downstream consequences of increased GzmB proteolytic activity on the choroidal ECM. GzmB is known to cleave several ECM components, including a major complement inhibitor, vitronectin.^26^ To confirm vitronectin as a substrate of GzmB in our experimental context, we performed an *in vitro* cleavage assay, which successfully demonstrated the generation of specific vitronectin cleavage fragments following incubation with active GzmB (Figure S1). Therefore, we investigated whether the high-risk CC microenvironment would exhibit a corresponding depletion of choroidal vitronectin, as a result of elevated GzmB secretion by RPE. To test this, we analyzed vitronectin immunoreactivity in genotyped non-AMD human donor eyes.

Quantitative reduction in inner choroid: immunofluorescence analysis of protective (homozygous TT) donor tissues revealed robust, vitronectin immunoreactivity along Bruch’s membrane and throughout the inner choroid—specifically the choriocapillaris and Sattler’s layer^34^ (red, Figures 2A and 2C). This suggests a preserved, functional complement-inhibiting ECM in TT eyes. In contrast, donor eye tissues from high-risk (homozygous CC) individuals exhibited significantly reduced vitronectin staining in these inner layers (red, Figures 2B and 2D). Quantification of choroidal vitronectin pixel intensity confirmed this observation (Figure 2E; *p* < 0.05).

**Figure 2:**
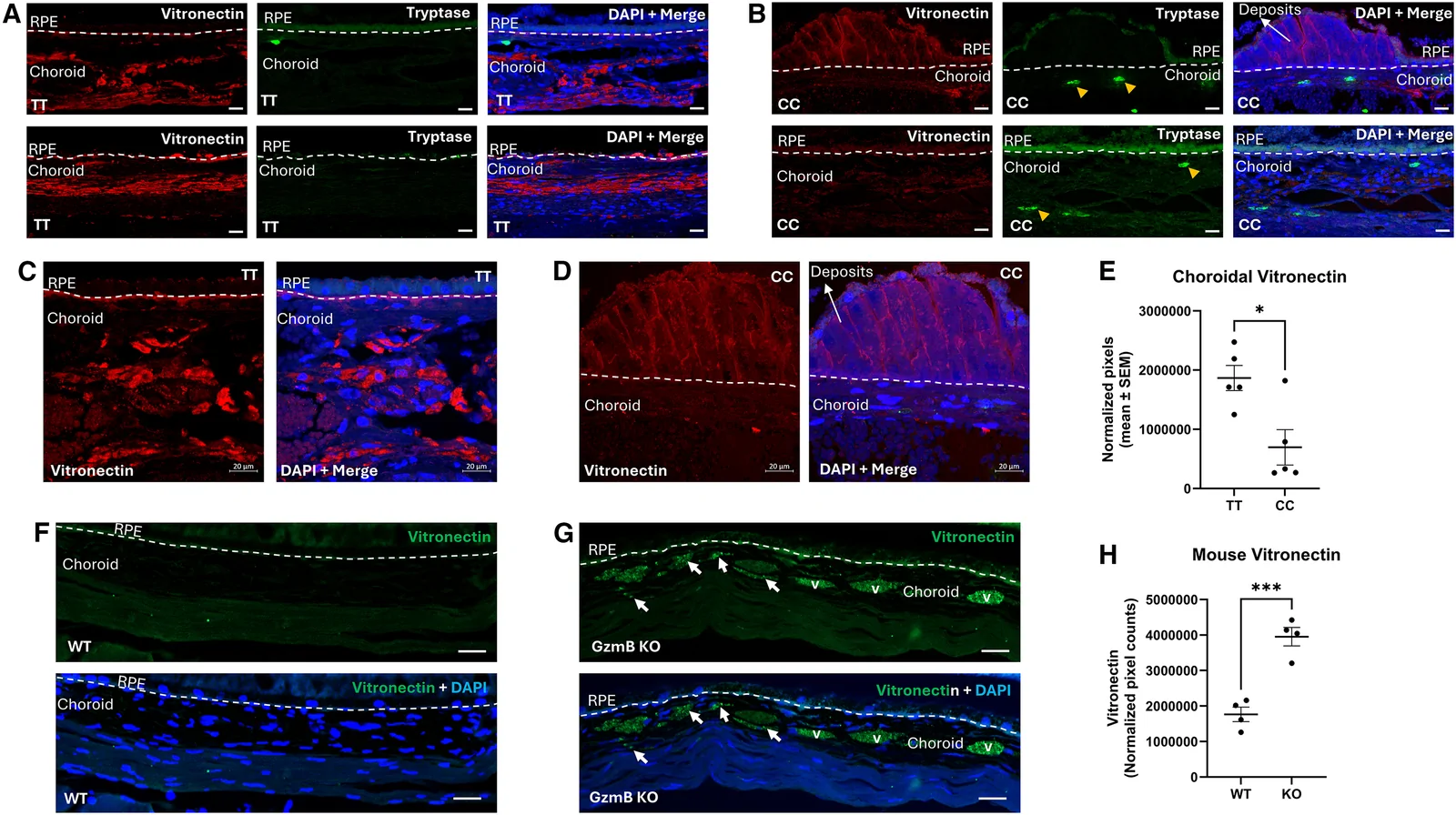
Loss of choroidal vitronectin observed in non-AMD, high-risk eyes, consistent with granzyme-B-mediated vitronectin turnover *in vivo* (A) Representative confocal images from two protective TT donors (*n*=5 independent eyes). Columns display vitronectin (red), mast cell tryptase (green), and the merged image with DAPI counterstain. Note the high levels of vitronectin immunoreactivity in the inner choroid and sparse mast cells. (B) Representative images from two high-risk CC donors (*n*=5 independent eyes), formatted as above. Vitronectin immunoreactivity (red) is reduced in the choroid, while mast cell density (green; orange arrowheads) is increased. Note that vitronectin in CC eyes is frequently concentrated within sub-RPE drusen-like deposits (diffuse blue signal within deposits represents drusen autofluorescence, not DAPI-stained nuclei; see Figure S9). For comparison, TT eyes exhibit fewer, smaller vitronectin-positive sub-RPE deposits consistent with normal aging (see Figure S10). (C and D) Higher magnification images of TT and CC eye cross sections showing localization of vitronectin (red) in the tissue. (E) Quantification of normalized pixel counts of vitronectin shows significantly reduced vitronectin immunoreactivity in inner choroid of CC eyes compared to TT eyes (*p* = 0.0144). (F) In WT mouse eye cross-sections (*n* = 4 animals), vitronectin immunoreactivity is generally low in choroid. (G) Strong vitronectin immunoreactivity (green) is observed in choroid of GzmB KO eyes (*n* = 4 animals). Note robust vitronectin signal within choriocapillaris (white arrows), as well prominent vitronectin associated with larger choroidal blood vessels (v). (H) Quantification of normalized pixel counts of vitronectin shows significantly increased vitronectin immunoreactivity in choroid of GzmB KO mouse eyes compared to WT (*p* = 0.0007). Human tissue sections in (A)–(D) were stained using a rabbit monoclonal anti-vitronectin antibody, while mouse tissue sections in (F) and (G) were stained using a rabbit polyclonal anti-vitronectin antibody. For quantification in (E) and (H), individual data points are displayed, with horizontal lines indicating the mean ± SEM. Statistical significance was determined using Mann-Whitney U test.

Qualitative redistribution of vitronectin: beyond quantitative reduction, the pattern of vitronectin deposition in high-risk eyes was also altered. While TT eyes exhibited occasional, small vitronectin-positive sub-RPE deposits consistent with normal age-related changes (Figure S10), vitronectin immunoreactivity in CC eyes was frequently concentrated between the sub-RPE space and Bruch’s membrane, concurrent with a reduction in overall inner choroidal levels (Figures 2B and 2D, arrows). This pattern aligns with an accelerated formation of early sub-RPE deposits and suggests that proteolysis may prevent vitronectin’s complement inhibition function while also generating aggregation-prone fragments. Furthermore, this reduction in inner-choroidal vitronectin was inversely associated with the presence of choroidal mast cells (tryptase+, green; Figure 2B, orange arrowheads), suggesting that mast-cell-derived proteases may contribute to cleavage of this complement regulator.

### GzmB regulates vitronectin turnover *in vivo*

To determine if GzmB actively regulates vitronectin turnover *in vivo*, we compared vitronectin immunoreactivity in cross-sections from 13- to 17-month-old GzmB knockout (KO) mice versus age-matched C57BL/6 wild-type (WT) controls, selecting this age group based on our previous finding of age-dependent GzmB accumulation in the outer retina.^24^ In WT mice with endogenous GzmB, vitronectin immunoreactivity was generally low and restricted to faint staining in the choroid (Figure 2F).

In contrast, choroidal tissue from GzmB KO mice exhibited robust vitronectin accumulation (Figure 2G). This prominent immunoreactivity was broadly distributed in the ECM of choriocapillaris (white arrows) and strongly associated with larger choroidal blood vessels (v), consistent with the known association of vitronectin with vascular basement membranes and smooth muscle cells.^35^ Quantification of pixel intensity confirmed significantly increased choroidal vitronectin levels in the GzmB KO choroid (Figure 2H; *p* < 0.001). Given that vitronectin is an abundant ECM component and plasma protein, its marked accumulation at these structural vascular sites in aged GzmB KO mice further indicates that GzmB plays a critical role in regulating local perivascular matrix turnover.

### Elevated MAC deposition in non-AMD, genetically at-risk eyes

To assess functional consequences of vitronectin loss, we examined the terminal complement pathway. Immunofluorescence revealed robust membrane attack complex (MAC/C5b-9) deposition along the choriocapillaris and RPE-Bruch’s membrane interface in high-risk CC donor eyes consistent across representative low-(20×) and high-magnification (40×) fields (Figures 3A and 3B). In contrast, protective TT eyes exhibited minimal, patchy MAC immunoreactivity (Figures 3C and 3D). Specific MAC immunoreactivity was confirmed by negative controls (Figures 3E and S2). Quantification confirmed significantly increased choroidal MAC in the high-risk CC group (Figure 3F; *p* < 0.05). These data align with previous studies demonstrating increased choroidal MAC in high-risk *CFH* genotypes^14^^,^^36^^,^^37^ and support the hypothesis that complement dysregulation due to GzmB-mediated proteolytic cleavage of inhibitors like vitronectin leads to unchecked MAC activation and choroidal tissue injury (Figure 6).

**Figure 3:**
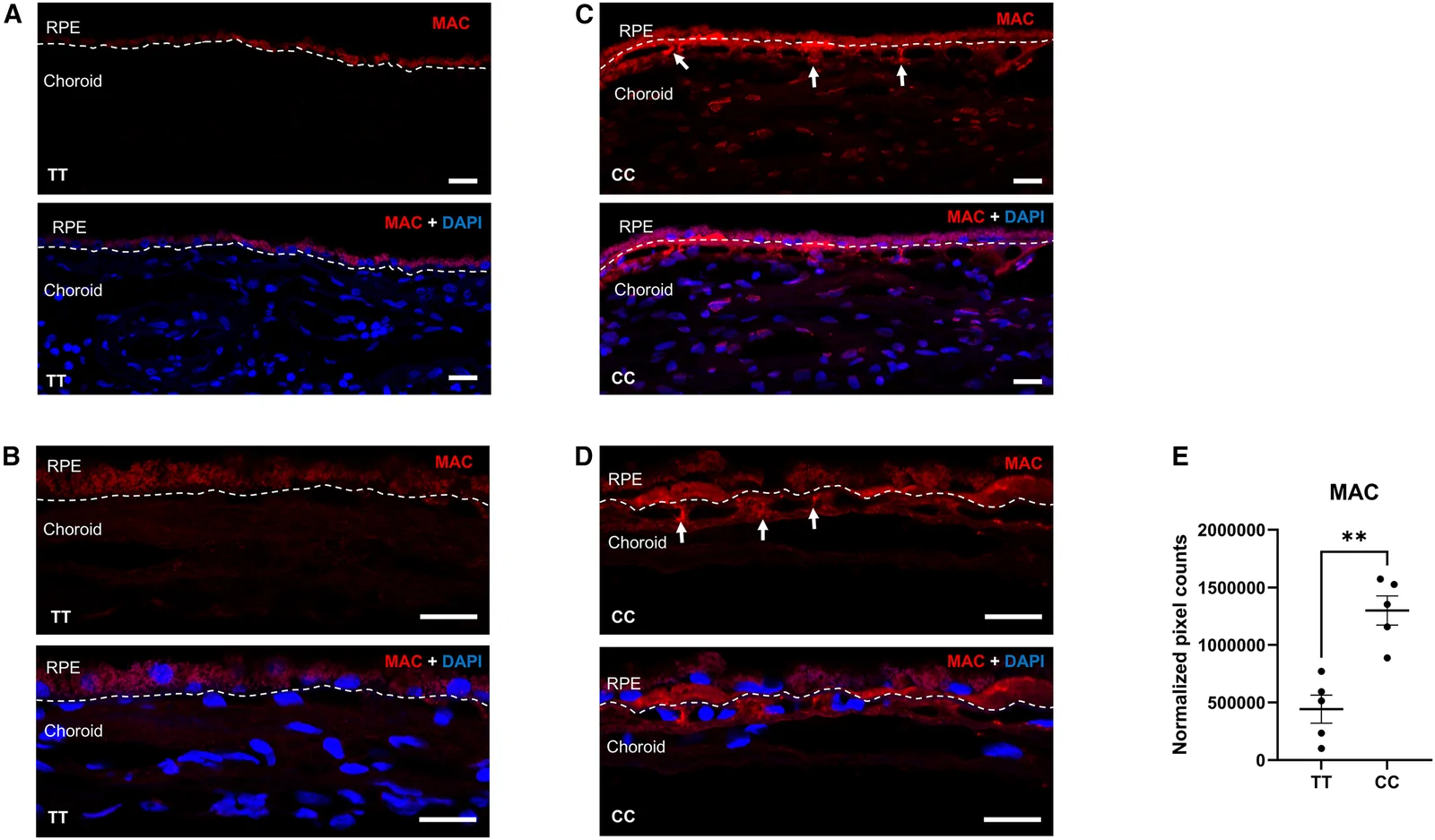
Elevated membrane attack complex deposition in non-AMD, high-risk eyes (A and B) Representative low- (20×) and high (40×)-magnification images showing minimal MAC immunoreactivity (red) in protective TT donor eyes (*n* = 5 independent eyes). (C and D) Representative low- (20×) and high (40×)-magnification images of high-risk CC donor eyes (*n* = 5 independent eyes), revealing prominent MAC deposits (red) accumulating around the choriocapillaris (white arrows) and on Bruch’s membrane. (E) Quantification of pixel intensity confirms significantly higher MAC deposition in CC as compared with TT eyes (*p* = 0.0079). For quantification in (E), individual data points are displayed, with horizontal lines indicating the mean ± SEM. Statistical significance was determined using Mann-Whitney U test.

### Complement product drives GzmB expression in RPE cells

Building on our observation of elevated choroidal C5a in high-risk *CFH* Y402H eyes,^38^ we investigated if C5a induces GzmB expression in RPE. Stimulating ARPE-19 cells with C5a (5 μg/mL; 24 h) significantly upregulated *GZMB* mRNA (Figure 4A; *p* < 0.05). Consistent with our findings of extracellular GzmB’s pro-inflammatory role,^22^^,^^39^ *TNF-α* (tumor necrosis factor α) (Figure 4B; *p* < 0.001) and *IL-8* (interleukin-8) (Figure 4C; *p* < 0.01) were upregulated, suggesting a pathogenic feedforward loop.^23^^,^^25^

**Figure 4:**
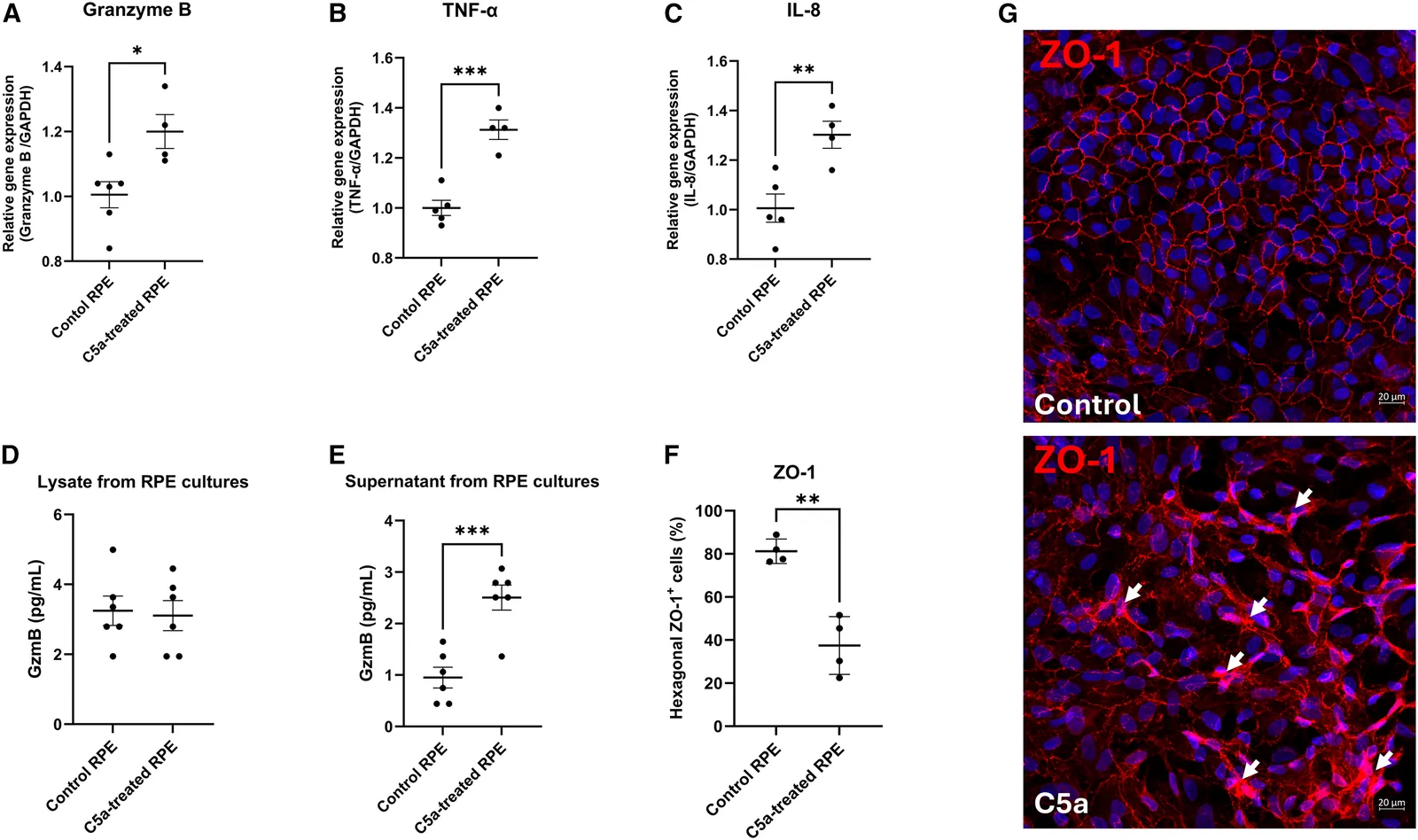
Complement fragment C5a drives granzyme B expression and tight junction disruption in retinal pigmented epithelial cells *(*A–C) *GZMB*, *TNFα*, and *IL8* gene expression in ARPE-19 cells significantly increased after exposure to 5 μg/mL of C5a for 24 hours compared to vehicle-treated controls (*p* = 0.024, *p* = 0.0007, and *p* = 0.007), indicating stimulation of a low-grade pro-inflammatory stress response. Relative gene expression was calculated using the 2^−ΔΔCt^ method; *n* = 4–6 biological replicates per group. (D and E) GzmB protein levels in RPE lysates remain stable but are significantly elevated in supernatants following C5a stimulation for 24 h (*n* = 6 biological replicates per group, *p*=0.0006). For panels (A)–(E), individual data points are displayed with horizontal lines indicating the mean ± SEM. (F) Quantification of percentage of cells displaying continuous, hexagonal membrane staining shows significant disruption in C5a-stimulated cells (*p* = 0.0037). Individual data points represent the mean percentage of intact cells per independent biological replicate (*n* = 4 per group). Horizontal lines indicate the mean ± SD. Statistical significance was determined using Welch’s *t* test. (G) Representative immunofluorescence of tight junction protein ZO-1 (red) and nuclei (blue). Note the continuous, honeycomb appearance of ZO-1 in vehicle-treated controls (top), indicative of an intact epithelial barrier. In contrast, RPE cells stimulated with C5a (48 h) exhibit marked tight junction disassembly, characterized by intracellular ZO-1 accumulation (bottom).

ELISA confirmed C5a stimulates GzmB secretion, while intracellular GzmB (lysates) remained unchanged (Figures 4D and 4E; *p* < 0.001), identifying RPE as a direct responder to C5a and a source of GzmB in the RPE-choroid microenvironment. Functionally, C5a stimulation (48 h) disrupted the continuous hexagonal ZO-1 barrier seen in controls (Figure 4G, top panel) and caused intracellular ZO-1 accumulation (Figure 4G, bottom panel). Quantification confirmed a significant reduction in the percentage of cells maintaining intact, hexagonal tight junctions (Figure 4F; *p* < 0.01). This mirrors our previous findings of GzmB-mediated ZO-1 cleavage, suggesting C5a-driven GzmB release compromises the outer blood-retinal barrier.^24^

### GzmB induces transcriptomic signatures of atrophy and cellular stress in RPE

Bulk RNA sequencing was performed on ARPE-19 cells. While we previously reported acute transcriptional changes at 6 h, here we focused on the 18-h time point to capture sustained gene expression patterns involved in chronic inflammation and ECM remodeling.^23^ To confirm the maintenance of RPE cell identity under our specific culture conditions, we first verified the stable baseline expression of RPE structural and functional markers, including *RBP1* (retinol-binding protein 1), *TJP1* (ZO-1), and *BEST1* (Bestrophin 1) across both control and GzmB-stimulated groups (Table S3). Subsequent analysis identified 1,464 differentially expressed genes (Figure 5A; *p-adj* < 0.05; fold change [FC] >1.5). Functional enrichment using Metascape (https://metascape.org)^40^ highlighted “*tissue morphogenesis*,” “*epithelial cell differentiation*,” and “*vasculature development*” (Figure 5B).

**Figure 5:**
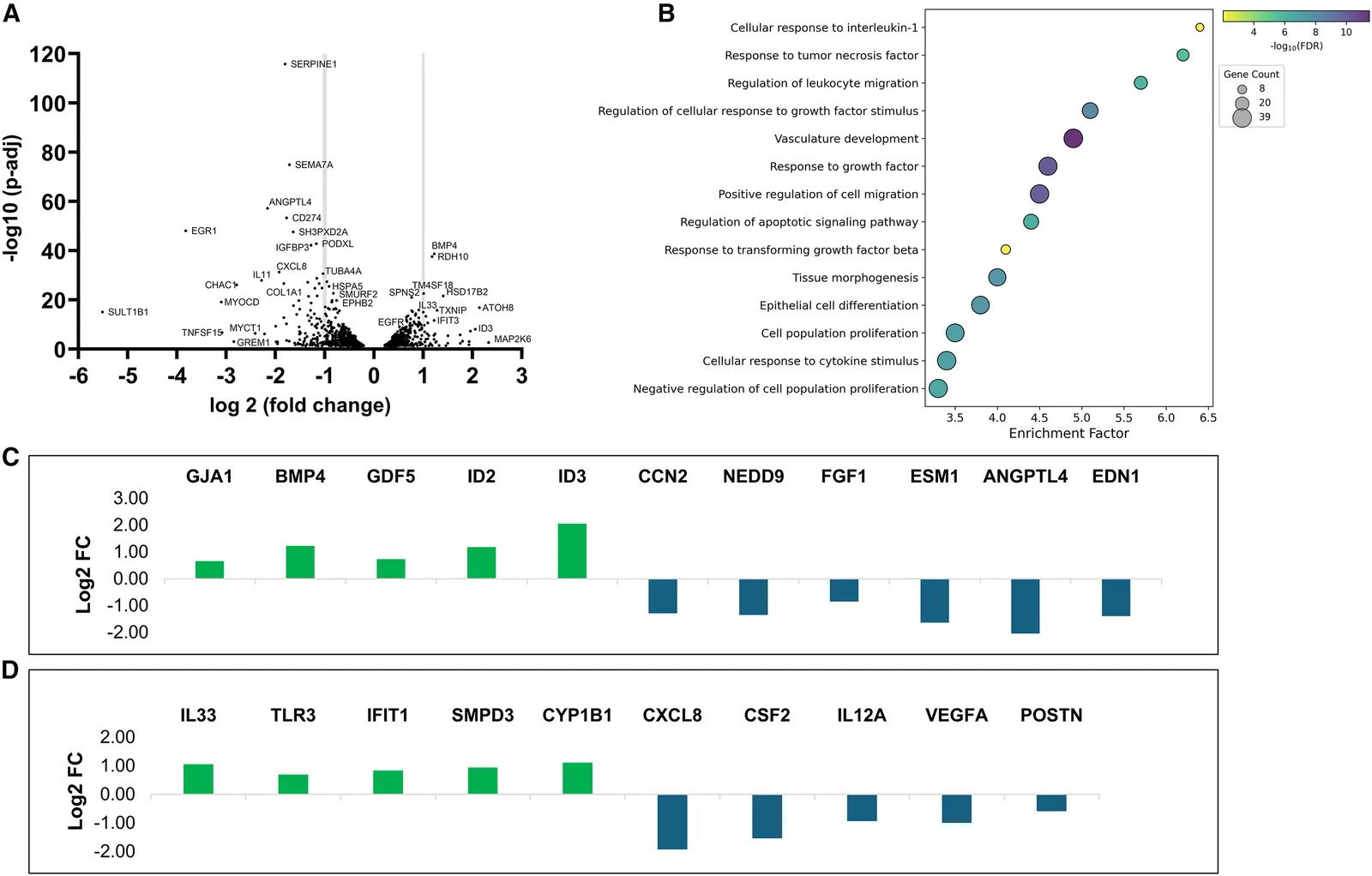
Granzyme B drives innate immune and cellular stress transcriptomic signatures in RPE cells (A) Volcano plot of differential gene expression in ARPE-19 cells stimulated with 100 nM GzmB (18 h) compared with controls. Vertical gray lines mark the 2-fold change threshold (log2 FC >1). (B) Bubble plot of selected enriched Gene Ontology (GO) biological processes relevant to AMD pathology. Circle size represents gene count; color indicates significance (-log10 FDR). (C and D) Fold change of key genes involved in epithelial cell fate, vascular support, and barrier integrity (C) or immune signaling, cellular stress, and trophic support (D). Green bars indicate significant upregulation, and blue bars indicate significant downregulation (>1.5-fold change). Data represent mean log2 FC (*n*=3, *p-adj* < 0.05).

Examination of individual genes revealed a complex response: a compensatory effort to reinforce the epithelial barrier was suggested by the upregulation of structural components (*GJA1* and *PPL*) and cell fate modulators (*BMP4* and *ID3*). Conversely, genes required for matrix signaling and vascular support were blunted (e.g., *CCN2*, *VEGFA*, and *EDN1*), indicating a loss of trophic support despite attempts to maintain identity (Figure 5C).

Pathway analysis also identified inflammatory signatures, revealing a distinct state of cellular stress: damage sensors (Toll-like receptor 3 [*TLR3*]) and alarmins (*IL-33*) were significantly upregulated alongside oxidative stress markers (*CYP1B1*). In contrast, broad pro-inflammatory and survival cytokines (*IL-8*, granulocyte-macrophage colony-stimulating factor [*GM-CSF*], and *IL-11*) were significantly downregulated (Figure 5D). Collectively, this profile indicates GzmB reprograms RPE toward a dysfunctional atrophic state, characterized by loss of immune homeostasis and barrier integrity (Figure 6).

**Figure 6:**
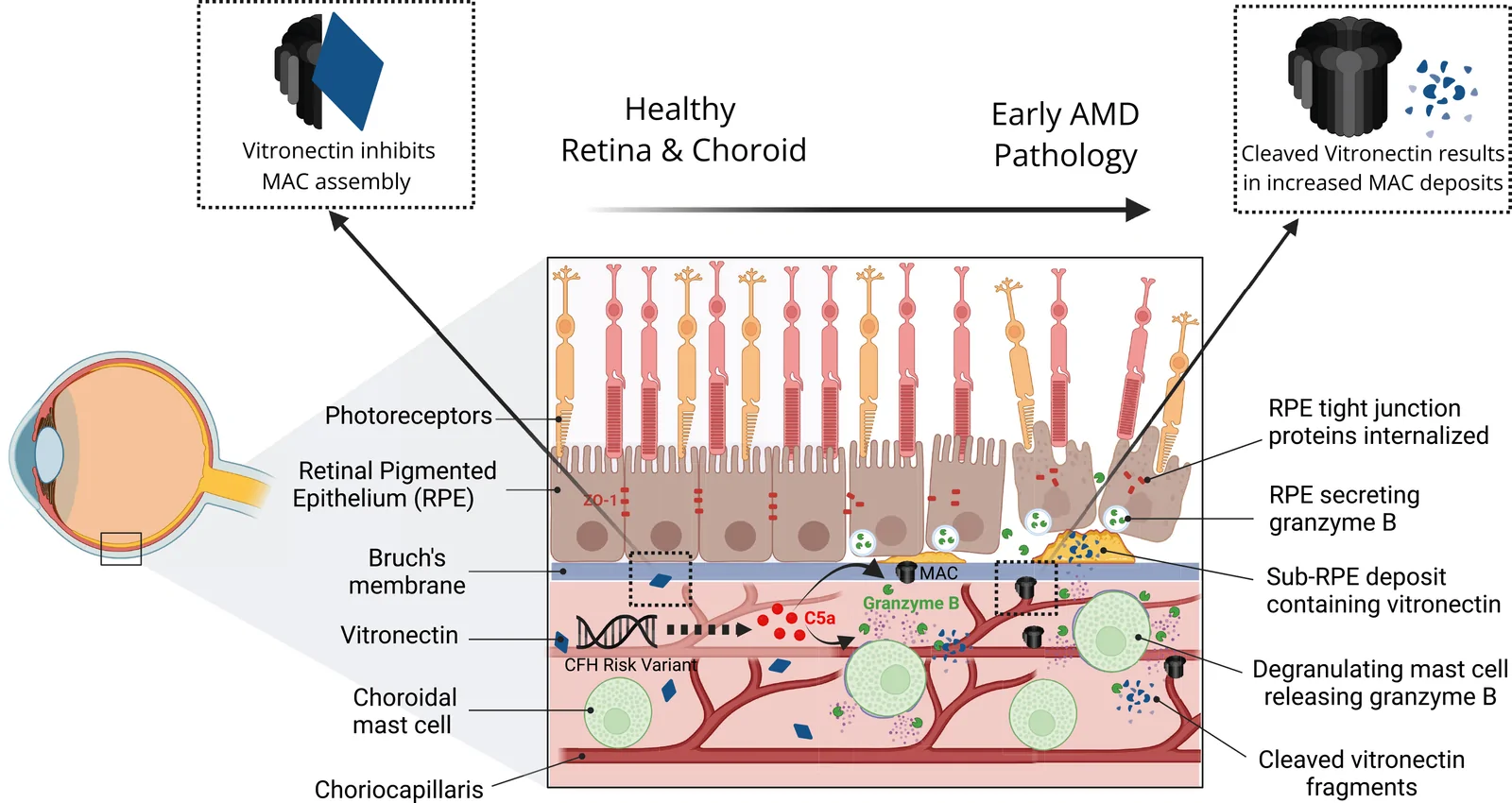
Schematic of the C5a-granzyme B-vitronectin axis in early AMD pathogenesis A magnified cross-sectional view of the human eye highlighting the retina-choroid interface illustrates the transition from healthy homeostasis (left) to early pathological remodeling (right) in AMD. *Healthy retina and choroid (left*): under physiological conditions, the retinal pigment epithelium (RPE) maintains robust barrier function, characterized by intact ZO-1 tight junctions. The underlying choroidal extracellular matrix (ECM) contains abundant full-length vitronectin, which acts as a potent complement inhibitor (magnified view) to protect the choriocapillaris and Bruch’s membrane. Choroidal mast cells are sparse and in a resting state. *Early AMD Pathology (right*): in eyes with *CFH* Y402H high-risk polymorphism, chronic local complement activation generates the anaphylatoxin C5a, which drives RPE cells to upregulate and secrete granzyme B (GzmB), disrupting ZO-1 junctional proteins and compromising the epithelial barrier. Choroidal mast cell infiltration further results in high concentrations of extracellular GzmB, leading to loss of choroidal vitronectin, and a pathogenic accumulation of vitronectin in sub-RPE deposits. The loss of functional vitronectin removes local complement inhibition (magnified view), resulting in unchecked membrane attack complex (MAC) deposition on Bruch’s membrane and choriocapillaris, driving early degenerative changes in AMD. Created in BioRender. Matsubara, J. (2026) https://BioRender.com/vtfv5f6.

## Discussion

The identification of the *CFH* Y402H polymorphism established the alternative complement pathway’s role in AMD; yet the downstream effectors remain unclear. Building on recent findings of mast cell infiltration into the inner choroid (concentrated near Bruch’s membrane) in early AMD,^31^ we examine the molecular cascade initiated by this genetic risk. While both AMD groups in our study exhibited elevated systemic GzmB compared to younger controls, we observed no systemic difference in GzmB levels between the high-risk and protective genotypes. This suggests that genetic risk acts locally within the tissue rather than causing late-stage systemic changes. Consistent with this, our earlier studies demonstrate that GzmB accumulates in the RPE and choroidal mast cells with age and soft drusen.^22^^,^^24^^,^^41^

The Y402H variant reduces the natural affinity of Factor H for polyanionic surfaces on the Bruch’s membrane, causing loss of complement inhibition. This results in chronic C5a generation, driving inflammation, cell recruitment, and cytokine release via C5aR-dependent signaling.^38^^,^^42^ C5a also contributes to inflammasome activation and proinflammatory cytokine production.^43^ We show that C5a strongly stimulates resident RPE to secrete pro-inflammatory cytokines and GzmB. These *in vitro* data are supported by our previous demonstration of C5a localization to the Bruch’s membrane and choroid in postmortem human eyes, placing it in direct proximity to drive GzmB expression we observe in the basal compartment of the RPE.^22^^,^^24^^,^^38^ We also observed increased mast cell recruitment in high-risk eyes, degranulating to release proteases like GzmB into the choroid. This aligns well with the established literature demonstrating that choroidal mast cells significantly accumulate and degranulate in human AMD, driving geographic atrophy through the release of matrix-degrading proteases such as tryptase.^44^^,^^45^^,^^46^ Our findings expand on this work by identifying GzmB as another critical complement-driven protease released by pathogenic mast cell activation. Previous work has also demonstrated that GzmB can cleave C5 to generate active C5a.^47^ This suggests a potential mechanism for sustaining chronic inflammation in high-risk eyes, where C5a-triggered GzmB release amplifies C5a production in a pathogenic feedforward loop.

While traditionally viewed as cytotoxic, our findings highlight the non-cytotoxic extracellular role of GzmB in ECM cleavage and immune regulation. We propose that a key pathogenic role of GzmB is the cleavage of ECM component vitronectin,^26^^,^^27^ typically produced by the RPE-choroid and found in ocular drusen.^48^ This GzmB-mediated cleavage of vitronectin and other ECM proteins promotes pathogenic ECM remodeling in multiple tissues, actively promoting cancer cell invasion, impaired skin wound healing, atherosclerosis, and rheumatoid arthritis.^26^^,^^49^^,^^50^^,^^51^ Beyond structural remodeling, this cleavage of vitronectin also disrupts a local immune checkpoint. Vitronectin normally regulates the alternative complement pathway by preventing membrane attack complex (MAC) assembly through its non-heparin-binding domains.^52^^,^^53^ Furthermore, as vitronectin is a known direct proteolytic substrate of GzmB,^26^ our data indicate that complement-driven secretion of GzmB actively cleaves vitronectin, thereby reducing local complement inhibition and leaving the tissue vulnerable to unchecked MAC deposition. Consequently, high-risk eyes with elevated GzmB exhibit depleted choroidal vitronectin and significant MAC deposition even without clinical AMD. While MAC forms lytic pores, sub-lytic MAC also promotes inflammation by triggering cytokine secretion and inflammasome activation.^54^^,^^55^

Previous studies show that complement activation upregulates vitronectin, suggesting a negative feedback mechanism to protect against tissue damage.^28^^,^^54^ Vitronectin exists in two forms *in vivo*.^56^ The predominant heparin-binding form in the ECM is prone to misfolding and amyloid-like aggregation at high local concentrations. Vitronectin also has high affinity for hydroxyapatite spherules in sub-RPE deposits, acting as a base for protein deposition in drusen.^19^^,^^57^ We and other investigators demonstrated that extracellular GzmB cleaves ECM components like laminin, fibronectin, and vitronectin.^22^^,^^24^^,^^26^^,^^58^ Here, we suggest that GzmB-mediated cleavage of vitronectin contributes to AMD by suppressing vitronectin’s complement-inhibiting function and generating pathogenic aggregation-prone vitronectin fragments in sub-RPE deposits (Figure 6).

Extracellular GzmB as a mediator of impaired vitronectin-complement inhibition represents a potential novel therapeutic target. Current therapeutic strategies for geographic atrophy focus mostly on complement components such as C3 or C5 inhibitors. While beneficial, these do not directly inhibit the protease activity cleaving natural complement regulators. GzmB inhibition could restore resistance to complement attack and prevent formation of amyloidogenic fragments by preserving the vitronectin reservoir. This is supported by SerpinA3N, a murine GzmB inhibitor, showing efficacy against neural toxicity and ECM damage in neuroinflammation, wound healing, and aortic aneurysm models.^59^^,^^60^^,^^61^ These findings suggest that GzmB inhibition is a viable therapeutic strategy for AMD.

In conclusion, we propose a molecular mechanism linking *CFH* genetic risk to early AMD structural pathology. We provide evidence that in non-AMD high-risk eyes, GzmB upregulation, consistent with C5a-mediated signaling, is associated with the loss of choroidal vitronectin and MAC deposition. GzmB acts as a critical mediator of retinal degeneration by initiating these localized early tissue changes. Therefore, therapies targeting extracellular GzmB offer a viable strategy for preserving retinal integrity and preventing AMD onset.

### Limitations of the study

A study limitation is using postmortem eye tissues, which represent a snapshot rather than disease progression. While *in vitro* models cannot fully replicate the ocular microenvironment, using C5a as a mediator is based on our observation of elevated C5a in *CFH Y402H* donor eyes,^38^ suggesting that this pathway described is active in early AMD. However, the precise molecular mechanism by which the *CFH Y402H* variant initiates the elevated C5a-GzmB axis remains unclear and warrants further investigation. A limitation of this study is that our sample sizes were insufficient to statistically evaluate the influence of biological sex on the results.

## Resource availability

### Lead contact

Requests for information and resources and reagents should be directed to and will be fulfilled by the lead contact, Dr. Joanne Matsubara (jms@mail.ubc.ca).

### Materials availability

This study did not generate new unique reagents.

### Data and code availability

• Bulk RNA-seq data have been deposited in GEO and are publicly available as of the date of publication. Accession numbers are listed in the key resources table.
• Original western blot and confocal imaging data have been deposited at Mendeley Data and will be publicly available as of the date of publication. The DOI is listed in the key resources table: https://data.mendeley.com/preview/p928vwvrxd?a=30b83715-2807-42fd-9cd6-e5af3644d3cb
• This study does not report original code.
• All other data supporting the findings are available within the article and supplemental information.

## Acknowledgments

This work was supported by the 10.13039/501100000038Natural Sciences and Engineering Research Council of Canada (10.13039/501100000038NSERC) (grant no. RGPIN 2018-04996); the Canadian Institutes of Health Research (10.13039/501100000024CIHR) (grant no. PJT-197949); Fighting Blindness Canada (grant no. AWD-7562-2024); and the 10.13039/501100008002Vancouver Coastal Health Research Institute (10.13039/501100008002VCHRI) (grant no. AWD-032288).

## Author contributions

H.C. and J.A.M. designed all the experiments and interpreted the results. H.C. performed human donor, animal, and cell culture experiments. Animal experiments were performed by J.X. and H.C. The bulk RNA sequencing was performed by R.B., A.H., and S.L.B. and analyzed by H.-S.Y. and H.C. General experimental contributions were made by H.-S.Y., B.L., and J.X. Data analyses were performed by H.C. and B.L. H.C. and A.B. wrote the manuscript. J.A.M. and D.J.G. obtained funding and critically revised the manuscript. All authors have read and approved the final manuscript.

## Declaration of interests

D.J.G. is a co-founder and serves as the Chief Scientific Officer of viDA Therapeutics.

## STAR★Methods

### Key resources table

**Table:** 

REAGENT or RESOURCE	SOURCE	IDENTIFIER
**Antibodies**		

Rabbit anti-Granzyme B polyclonal antibody	Abcam	ab4059; RRID: AB_304251
Mouse anti-Tryptase monoclonal antibody	Abcam	ab2378; RRID: AB_303023
Rabbit anti-Vitronectin polyclonal antibody- used in mouse tissue sections	Abcam	ab272865
Rabbit anti-Vitronectin monoclonal antibody- used in human tissue sections	Abcam	ab45139; RRID: AB_778872
Mouse anti-C9 neoantigen monoclonal antibody	Hycult Biotech	HM2264; RRID: AB_10502672
HRP-conjugated Goat Anti-Rabbit antibody	ThermoFisher Scientific	31460; RRID: AB_228341
Rabbit anti-ZO-1 polyclonal antibody	Invitrogen	61-7300; RRID: AB_2533938
Alexa Fluor 647 AffiniPure Donkey Anti-rabbit	Jackson ImmunoResearch	711-605-152; RRID: AB_2492288
Alexa Fluor 488 Goat Anti-rabbit	ThermoFisher Scientific	A-11070; RRID: AB_2534114
Alexa Fluor 546 Goat Anti-mouse	ThermoFisher Scientific	A-21123; RRID: AB_2535765
DAPI nuclear stain	Invitrogen	D1306

**Biological samples**		

Human plasma samples (dry AMD patients and controls)	Eye Care Center at Vancouver General Hospital (VGH)	The UBC Retina and Vitreous Service
Human donor eyes (non-AMD, *CFH* genotyped)	Eye Bank of British Columbia	https://www.vch.ca/en/service/eye-bank-british-columbia

**Chemicals, peptides, and recombinant proteins**		

Recombinant human C5a	R&D Systems	2037-C5-025
Recombinant human Granzyme B	Bon Opus Biosciences	www.bonopusbio.com
Recombinant human vitronectin	Thermo Fisher (Peprotech)	AF-140-09-100UG
Paraformaldehyde (PFA), 4% solution	Fisher Scientific	50-980-494
Triton X-100	Sigma-Aldrich	9036-19-5
Normal goat serum (NGS)	Vector Laboratories	S-1000-20
Mowiol 4-88 mounting medium	MilliporeSigma	475904100GM
Tween 20	Fisher Scientific	BP337-500
Blocker™ FL Fluorescent Blocking Buffer	Thermo Fisher Scientific	37565
SuperSignal™ West Atto Ultimate Sensitivity Substrate	ThermoFisher Scientific	A38555

**Critical commercial assays**		

Human Granzyme B ELISA Kit	Thermo Fisher Scientific	BMS2027-2
RNeasy Mini Kit	Qiagen	Cat# 74104
High Capacity cDNA Reverse Transcription Kit	Applied Biosystems	Cat# 4368814
BCA Protein Assay Kit	Thermo Fisher Scientific	Cat# 23225

**Deposited data**		

Raw confocal microscopy and western blot data	Mendeley Data	https://data.mendeley.com/preview/p928vwvrxd?a=30b83715-2807-42fd-9cd6-e5af3644d3cb
Bulk RNA-seq Data	Gene Expression Omnibus (GEO)	Accession Number: GSE322497

**Experimental models: Cell lines**		

Human retinal pigment epithelial cells (ARPE-19)	ATCC	CRL-2302

**Experimental models: Organisms/strains**		

Mouse: C57BL/6J	Jackson Laboratory	JAX: 000664; RRID: IMSR_JAX:000664
Mouse: Granzyme B knockout (Gzmb−/−, C57BL/6J background)	Jackson Laboratory	*B*6.129*S*2-*Gzmb*^*tm*1*Ley*^/*J*

**Oligonucleotides**		

IL-8 Primers IL-8 Fwd: TAGCAAAATTGAGGCCAAGG IL-8 Rev: GGACTTGTGGATCCTGGCTA	Integrated DNA Technologies	N/A
TNF-α Primers TNF-α Fwd: TCCTTCAGACACCCTCAACC TNF-α Rev: AGGCCCCAGTTTGAATTCTT	Integrated DNA Technologies (IDT)	N/A
Granzyme B Primers GAPDH Fwd: CGACCACTTTGTCAAGCTCA GAPDH Rev: AGGGGTCTACATGGCAACTG	Integrated DNA Technologies (IDT)	N/A

**Software and algorithms**		

ImageJ	National Institutes of Health	https://imagej.nih.gov/ij/
GraphPad Prism v10.3.1	GraphPad Software	https://www.graphpad.com
STAR aligner (v2.6.0a)	Dobin et al.^62^ Bioinformatics, 2013	https://github.com/alexdobin/STAR
HTSeq (v0.11.2)	Anders et al.^63^ Bioinformatics, 2015	https://htseq.readthedocs.io
DESeq2 (v1.16.1)	Love et al.^64^ Genome Biology, 2014	https://bioconductor.org
MultiQC	Ewels et al.^65^ Bioinformatics, 2016	https://multiqc.info
Metascape	Zhou et al.^40^ Nature Communications, 2019	https://metascape.org
Python (v3.9; Pandas, Seaborn, Matplotlib)	Python Software Foundation	https://www.python.org
iBright Analysis Software	Thermo Fisher Scientific	https://www.thermofisher.com/ca/en/home/technical-resources/software-downloads/ibright-western-imager.html
Zen 2.6 Blue	Carl Zeiss	https://www.zeiss.com/microscopy/en/products/software/zeiss-zen.html

**Other**		

Zeiss LSM 800 confocal microscope	Carl Zeiss	https://www.zeiss.ca/corporate/en/home.html
Zeiss LSM 880 Airyscan confocal microscope	Carl Zeiss	https://www.zeiss.ca/corporate/en/home.html
DNBSEQ-G400RS sequencer	Complete Genomics	https://www.completegenomics.com/media/css-00058-system-guide-dnbseq-g400rs-rev-c.pdf
TapeStation 4200 system	Agilent Technologies	https://www.agilent.com/en/product/automated-electrophoresis/tapestation-systems/tapestation-instruments/4200-tapestation-system-228263?srsltid=AfmBOoofq8SpQ30_sB3doAI-OIjXAlMTMltDe5lCl5_WPgcGbNfvlYDQ
iBright FL1500 Imaging System	Thermo Fisher Scientific	https://www.thermofisher.com/order/catalog/product/A44115?SID=srch-srp-A44115

### Experimental model and study participant details

#### Human participants and plasma samples

Human plasma samples (Cohort 1) were obtained from patients with clinically confirmed bilateral dry age-related macular degeneration (AMD; age ≥55 years) (*n* = 16) and a separate cohort of younger healthy controls (*n* = 7). Controls were not age-matched (mean age 46.7 ± 3.8 years, SEM) and were included as a physiological baseline for systemic granzyme B levels rather than for direct statistical comparison.

AMD plasma samples were further stratified by *CFH* Y402H genotype (CC, TT, CT) as summarized in Table S2. Overall cohort demographics included a mean age of 75.6 ± 2.8 years with 70% female and 30% male participants.

Inclusion criteria required confirmed bilateral dry AMD. Exclusion criteria included neovascular AMD, geographic atrophy, active malignancy, recent cardiovascular complications, uncontrolled diabetes, or use of medications that could confound inflammatory or protease-related outcomes. Plasma samples were collected and stored at −80°C until analysis.

All studies involving human participants were conducted in accordance with the Declaration of Helsinki and approved by the Providence Health Care and University of British Columbia Clinical Research Ethics Boards (H20-02944). Written informed consent was obtained from all participants.

#### Human donor eyes

Postmortem human donor eyes (Cohort 2; non-AMD) were obtained from the Eye Bank of British Columbia and genotyped for the *CFH* Y402H polymorphism. Donors were stratified into homozygous high-risk (CC; *n* = 5) and protective (TT; *n* = 5) groups.^66^ All tissues were collected and preserved within 22 hours postmortem using standardized protocols established in our laboratory and prior studies.^24^^,^^37^^,^^66^ Exclusion criteria included infection, central nervous system pathology, or intrinsic ocular disease. Donor groups were age-matched, with no significant difference between TT (62.6 ± 4.0 years, SEM) and CC (61.0 ± 5.3 years, SEM) cohorts (Table S1). Sex distribution was as follows: TT (60% male, 40% female) and CC (80% male, 0% female, 20% unknown).

Eyes were fixed in 10% buffered formalin and processed into 6 μm paraffin-embedded sections. For each donor, contralateral globes were used for histological analysis. Previous work has established that granzyme B localizes predominantly to the central macula.^24^ As the current study focuses on molecular mechanisms of extracellular matrix degradation and complement activation, retinal sections were obtained from multiple regions of the posterior pole. These mechanisms are not expected to vary by retinal location; however, the lack of strict macular localization is acknowledged as a limitation. Additional donor demographics are provided in Tables S1 and S2.

#### Cell lines and culture conditions

ARPE-19 human retinal pigment epithelial cells (ATCC CRL-2302) were purchased from American Type Culture Collection (CRL-2302; ATCC, Manassas, VA, USA). Cells were obtained at Passage 19 (based on the company’s lot information) and cultured under standard conditions in DMEM/F12 supplemented with 10% fetal bovine serum and 1% penicillin/streptomycin. ARPE-19 cell identity was authenticated transcriptomically by verifying stable baseline expression of RPE markers (Table S3), and phenotypically via ZO-1 tight junction formation (Figure 4G). The cell lines were not tested for mycoplasma contamination. For experiments, cells (P22-25) were seeded onto chamber slides or 12-well plates depending on downstream applications and serum-starved overnight (2% FBS). Cells were stimulated with recombinant human C5a (5 μg/mL, reconstituted in sterile PBS to a stock concentration of 100 μg/mL prior to use.) for 24-48 hours or recombinant human granzyme B (100 nM) for 16-18 hours. All control wells were treated with an equivalent volume of sterile PBS (vehicle control). Supernatants and cell lysates were collected and stored at −80°C for downstream analysis. For stimulation, cells were seeded in Lab-Tek chamber slides (Thermo Scientific) or 12-well plates (Corning) and serum-starved (2% serum) overnight. Experimental conditions included stimulation with recombinant human C5a (5 μg/mL, 24-48 hours) or recombinant Granzyme B (100 nM, 16-18 hours). Cells were seeded on chamber slides or 12-well plates depending on downstream applications. Supernatants and lysates were collected and stored at -80°C.

#### Animals

C57BL/6J wild-type (WT) and granzyme B knockout (Gzmb−/−) mice were used. Gzmb−/− mice were generated by targeted deletion of exon 1 of the *GzmB* gene and replacement with a PGK-neo cassette to establish germline-transmitting mice on a C57BL/6J background.^67^ This germline deletion results in complete systemic loss of GzmB expression, including in the choroidal tissue. WT mice were female, while Gzmb−/− mice included both sexes (2 males, 2 females), aged 13-17 months. Mice were housed under standard conditions with controlled light-dark cycles and *ad libitum* access to food and water. Group allocation was performed randomly. Eyes were enucleated, fixed in 10% buffered formalin, and processed into 6 μm paraffin sections. All animal procedures were approved by the University of British Columbia Animal Care Committee (A24-0098) and conducted in accordance with Canadian Council on Animal Care and ARVO guidelines.

### Method details

#### Immunohistochemistry and immunocytochemistry

Retinal sections underwent citrate buffer antigen retrieval, followed by blocking with 5% normal goat serum and permeabilization using Triton X-100. Sections were incubated overnight at 4°C with primary antibodies against Granzyme B, Tryptase, Vitronectin, and MAC, followed by fluorescent secondary antibodies. Nuclei were counterstained with DAPI and mounted. For *in vitro* junctional integrity analysis, ARPE-19 cells were stained using an anti-ZO-1 antibody. Antibody specificity was validated using multiple approaches: Negative controls involving omission of primary antibodies (Figures S2, S3, and S4) were used to establish background fluorescence. Biological validation of the granzyme B and tryptase antibodies was confirmed via immunofluorescence on human tonsil sections, which served as positive control exhibiting the expected labeling (Figures S5 and S6). Specificity was also validated using Western blot, confirming the expected molecular weight bands for both granzyme B and vitronectin (Figures S7 and S8). Additionally, MAC, ZO-1, and tryptase antibodies have been extensively validated in the literature.

#### Confocal microscopy and image analysis

Confocal imaging was performed on a Zeiss LSM 800 confocal microscope (20x/40x) using Zen 2.6 Blue software (Carl Zeiss, Germany). Super-resolution imaging of mast cell granules used a Zeiss LSM 880 Airyscan. Acquisition settings were kept constant across experimental groups to enable quantitative signal intensity comparison. Quantification was restricted to the choroid and sub-RPE deposits to exclude variable RPE autofluorescence. For each donor, 8-10 random fields (20×) were analyzed and GzmB+ and Tryptase+ cells were manually counted. Co-expression was calculated as the percentage of total Tryptase+ cells that were also positive for GzmB. Vitronectin and MAC immunofluorescence were quantified by normalizing positive pixel counts to inner choroid area (pixels/mm2). The percentage of ARPE-19 cells exhibiting continuous, hexagonal membrane staining was calculated for each field, and these values were averaged to generate a single mean data point per independent biological replicate. All analyses were conducted in a blinded manner.

#### ELISA

Granzyme B levels were quantified using a commercial ELISA kit according to manufacturer instructions.

#### Quantitative PCR

Total RNA was extracted, reverse-transcribed into cDNA, and gene expression quantified using real-time PCR for Granzyme B, IL-8, and TNF-α.

#### RNA sequencing and bioinformatics

RNA integrity was confirmed (RIN >9). Libraries were prepared and sequenced (paired-end, 100 bp). Reads were aligned to hg38 using STAR, quantified using HTSeq, and analyzed using DESeq2. Significance thresholds: adjusted *p* < 0.05, fold change > 2. Functional enrichment analysis was performed using Metascape.

#### Western blot

To assess the expression and/or cleavage of vitronectin and GzmB, Western blots were performed. Proteins were run on a 10% SDS-PAGE gel and transferred to a PVDF membrane. Membranes were blocked in Blocker FL Fluorescent Blocking Buffer for 1 hour at room temperature before probing with the primary antibodies at a dilution of 1:1000 vitronectin (ab45139) or GzmB (ab4059) in Blocker FL Fluorescent Blocking Buffer in 0.1% Tween 20 overnight at 4 °C. Blots were then probed with rabbit IgG HRP-conjugated secondary antibody (1:100,000 dilution) in Blocker FL Fluorescent Blocking Buffer in 0.1% Tween 20 for 2 h at RT. All membrane washes were performed with 0.2% Tween 20 in PBS. The respective proteins were detected with SuperSignal West Atto Ultimate Sensitivity Substrate. Membranes were imaged with iBright FL1500 Imaging System and bands were analyzed using iBright Analysis Software.

#### Vitronectin cleavage assay

To evaluate proteolytic cleavage of vitronectin by GzmB, an *in vitro* assay was performed. Recombinant human vitronectin (200 ng) was incubated with 100 nM recombinant human GzmB in a total volume of 20 μL in PBS. Negative control contained 200 ng of vitronectin in PBS without the addition of GzmB. The reactions were carried out in low-protein-binding tubes and incubated at 37°C for 1 hour. Following incubation, the enzymatic reactions were terminated by the addition of a 6X loading buffer containing β-mercaptoethanol. The samples were then denatured by boiling at 95°C for 5 minutes. The resulting cleavage products were resolved via SDS-PAGE, transferred to a membrane, and analyzed by Western blotting using anti-vitronectin antibody (ab45139).

### Quantification and statistical analysis

Statistical analyses were performed using GraphPad Prism v10. Normality assessed using Shapiro-Wilk test. Parametric: unpaired *t*-test (Welch correction). Non-parametric: Mann-Whitney U test. Data expressed as mean ± SEM. Statistical significance defined as *p* < 0.05.
