# Supplementary material for: Factor H polymorphism elevates granzyme B and impairs complement inhibition in human and mouse models
Source: iScience. 2026 Oct 19;29(11):117957. doi: 10.1016/j.isci.2026.117957 (PMC13695112; doi:10.1016/j.isci.2026.117957)
Supplement: Supplementary file 1 [file mmc1.pdf]

iScience, Volume *29*

Supplemental information

Factor H polymorphism elevates granzyme B and impairs complement inhibition in human and mouse models

Harshini Chakravarthy, Jeanne Xi, Hyung-Suk Yoo, Andrew Buss, Boaz Li, Robert Bell, Anne Haegert, Stephane Le Bihan, David J. Granville, and Joanne A. Matsubara

Figure S1. ***In vitro* cleavage of vitronectin by granzyme B.** Western blot demonstrating proteolytic cleavage of human vitronectin by active granzyme B (GzmB). Recombinant human vitronectin was incubated either alone (left lane) or with active GzmB for 1 hour (right lane). The untreated sample shows only the intact, full-length vitronectin protein, while incubation with GzmB results in generation of a lower molecular weight cleavage fragment. The red box and red arrow highlight the appearance of this cleavage product only in the GzmB-treated lane, confirming vitronectin as a substrate for GzmB.

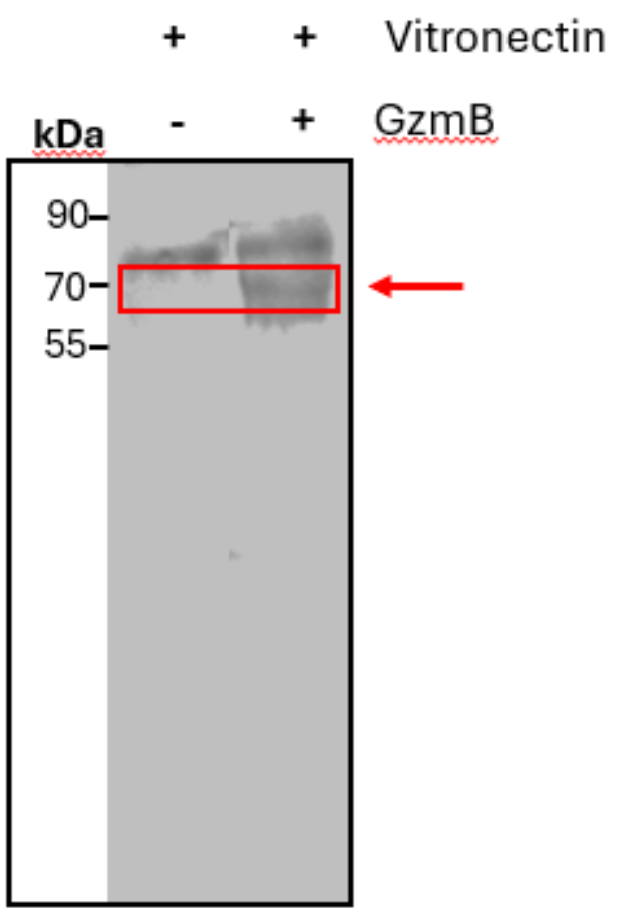

Figure S2. Negative control for MAC immunofluorescence. Representative image of a human RPE/choroidal section (CC) where primary antibody was omitted. The absence of immunoreactivity in the choroid in the red channel (MAC; 546 nm) confirms specificity of this antibody. Variable autofluorescence was observed in RPE layer and was excluded from image analysis. Nuclei are counterstained with DAPI (blue). Scale bar = 20µm.

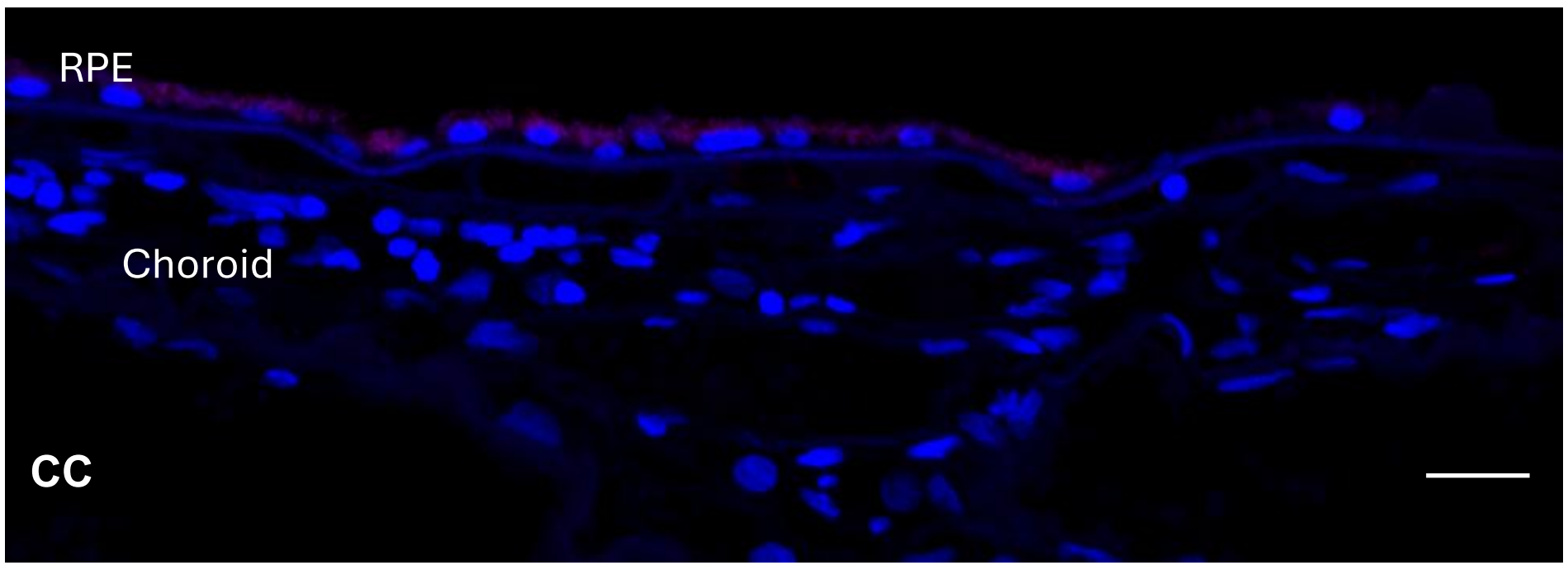

Figure S3. Negative controls for granzyme B and tryptase immunofluorescence.

(A) Negative control for granzyme B (GzmB). Representative merged image where the primary antibody for GzmB was omitted. The presence of tryptase-positive mast cells (red) confirms successful staining in the red channel, while the absence of specific green signal (except for characteristic RPE autofluorescence) confirms the specificity of the GzmB antibody.

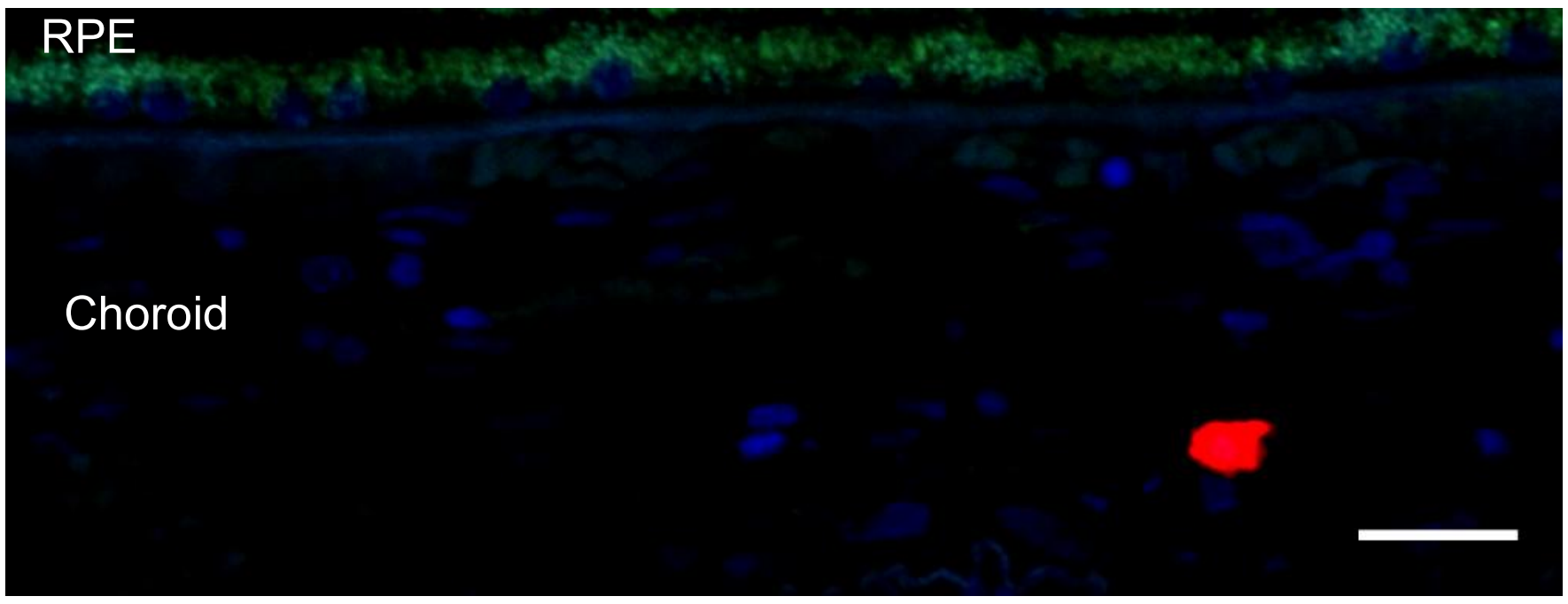

(B) Negative control for tryptase. Representative merged image where the primary antibody for tryptase was omitted. GzmB-positive cells (green) are clearly visible, confirming successful staining in the green channel. The absence of signal in the red channel confirms the specificity of the tryptase antibody. Nuclei are counterstained with DAPI (blue). Scale bars = 20 μm.

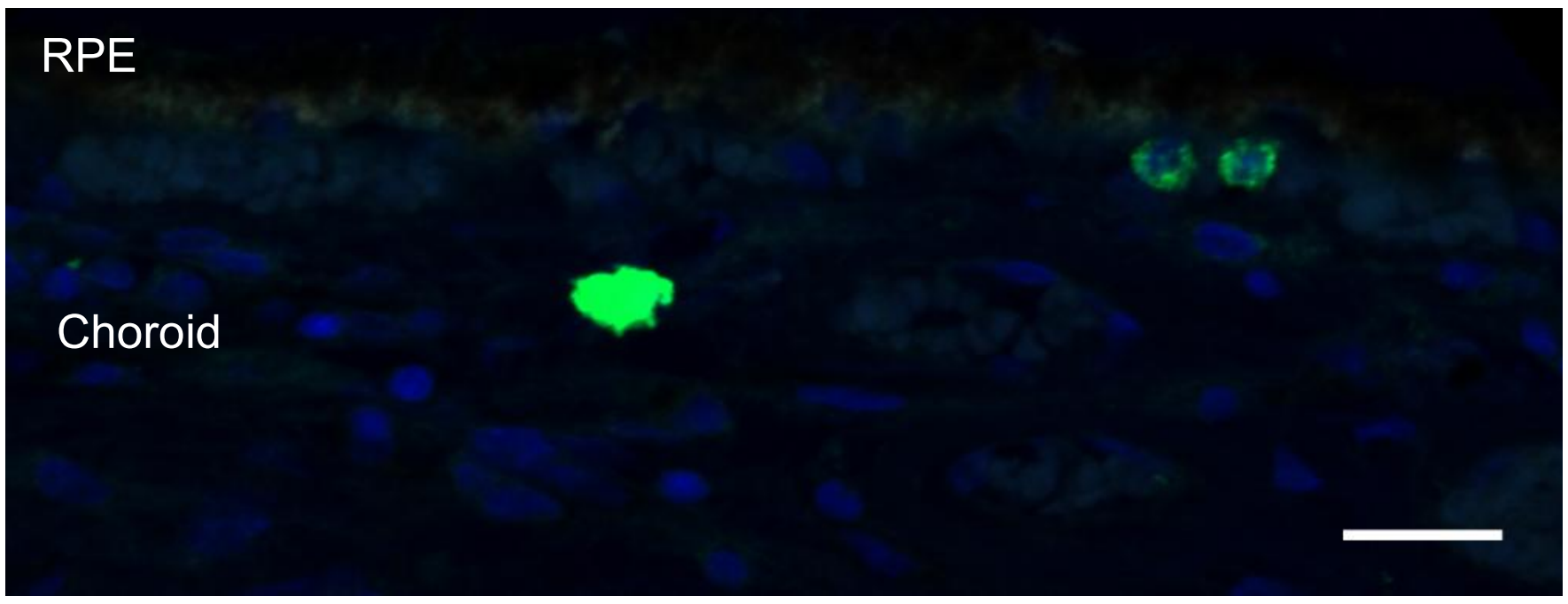

Figure S4. Negative control for vitronectin and tryptase immunofluorescence. Representative merged image of a human RPE/choroidal section where primary antibodies were omitted. The absence of immunoreactivity in the red channel (Vitronectin; 647 nm) and green channel (Tryptase; 546 nm) in the choroid confirms the specificity of these antibodies. Nuclei are counterstained with DAPI (blue). Scale bar = 20μm.

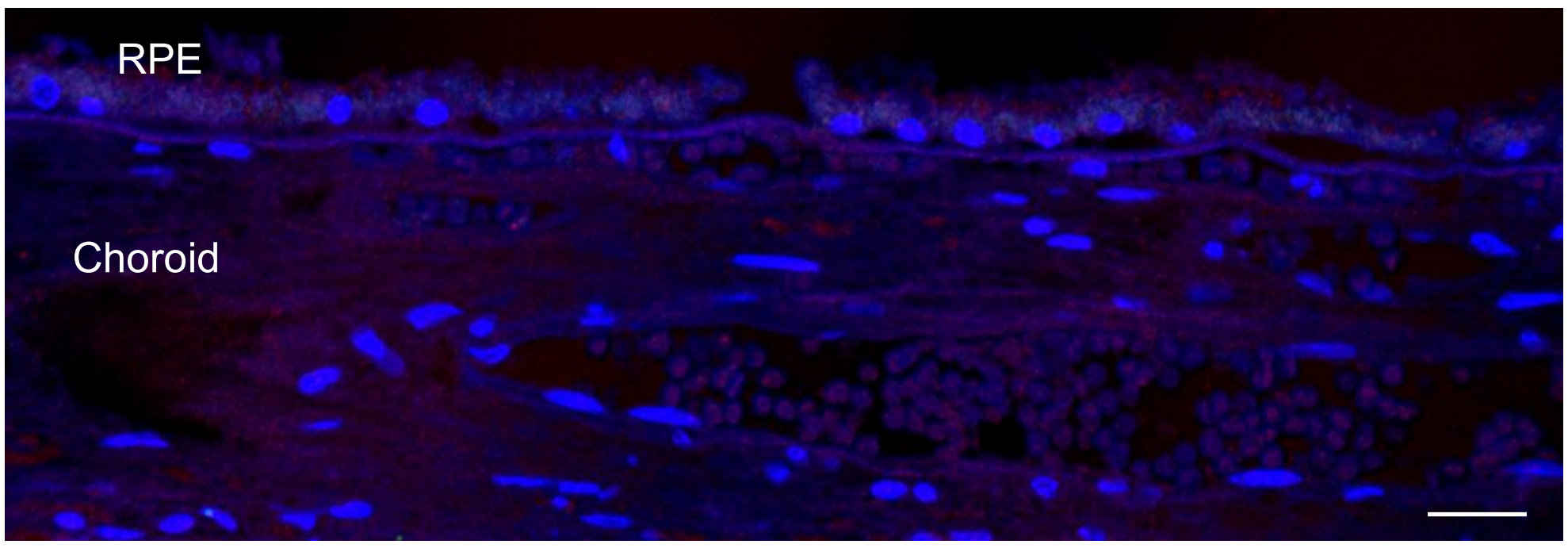

Figure S5. Biological validation of Granzyme B Antibody specificity. Immunofluorescence imaging of human tonsil tissue, used as a biological positive control. *Top*: Merged image shows Granzyme B-positive immune cells in green, with white arrows highlighting representative examples, with nuclei counterstained by DAPI (blue). *Bottom*: The corresponding single-channel image for Granzyme B (green) confirms specific labeling, with arrows tracking the same representative cells shown in the merged panel above.

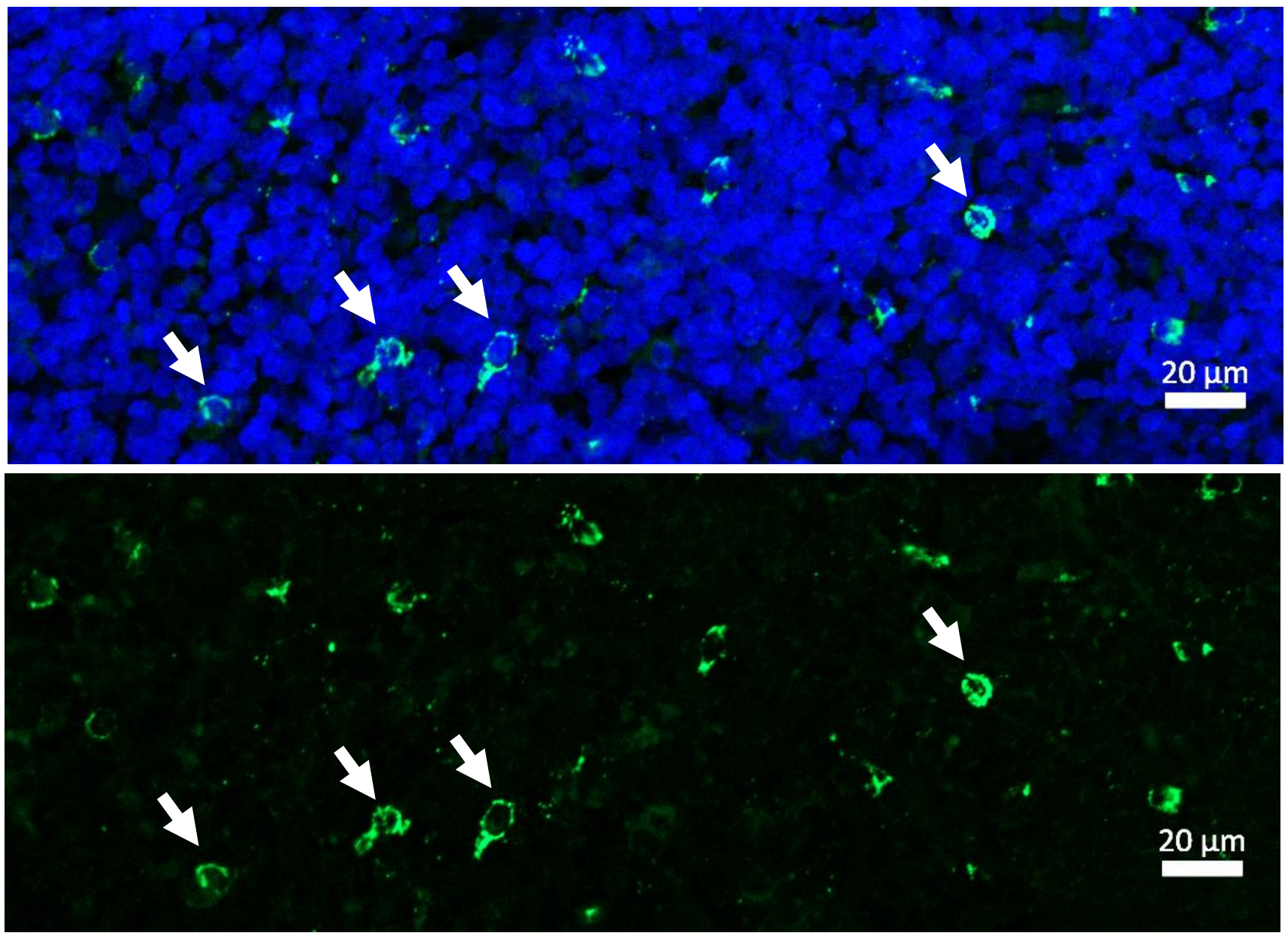

Figure S6. Biological validation of Tryptase and Granzyme B dual immunolabeling. Dual-immunofluorescence imaging of human tonsil tissue to confirm antibody compatibility and specificity. *Top*: Merged image demonstrates colocalization of Granzyme B (green) and Tryptase (red) within resident mast cells (white arrows). Nuclei are counterstained with DAPI (blue). *Bottom*: The corresponding single-channel image for Tryptase (red) confirms the presence of the specific mast cell marker, with white arrows pointing to the cells observed in the merged panel above. Scale bar = 20µm.

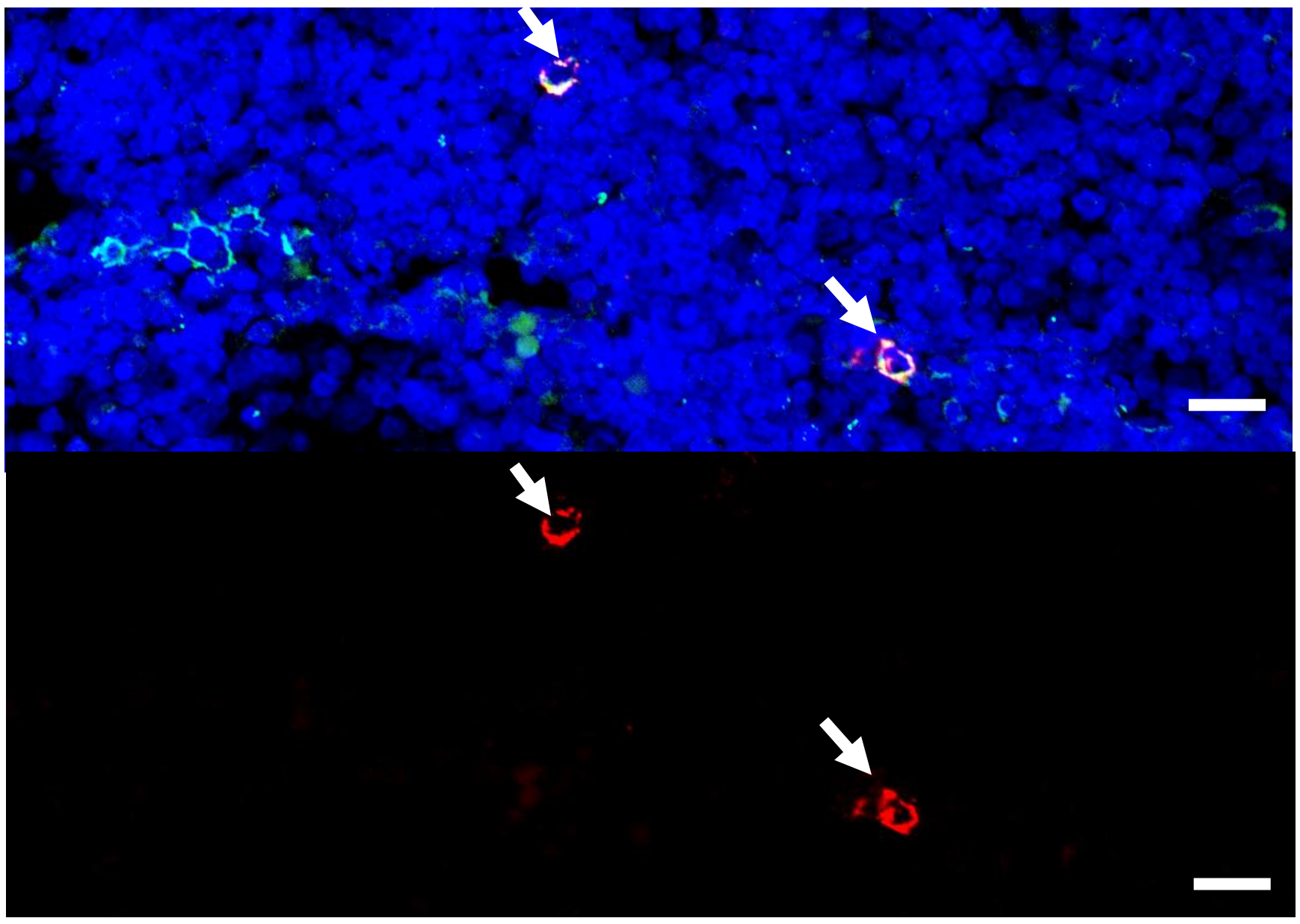

Figure S7. Validation of granzyme B antibody specificity. Western blot demonstrating specificity of the anti-granzyme B primary antibody (Abcam, ab4059). *Left panel:* Recombinant human granzyme B protein was used as a positive control. The antibody detects a specific band at ~35 kDa (red arrow), which corresponds to the expected molecular weight. *Right panel*: Western blot of whole-cell lysate from YT cells (human NK cell line) probed with the same antibody, detects a specific band at ~70 kDa (red arrow), corresponding to the stable Granzyme B-SerpinB9 (intracellular GzmB inhibitor) covalent complex.

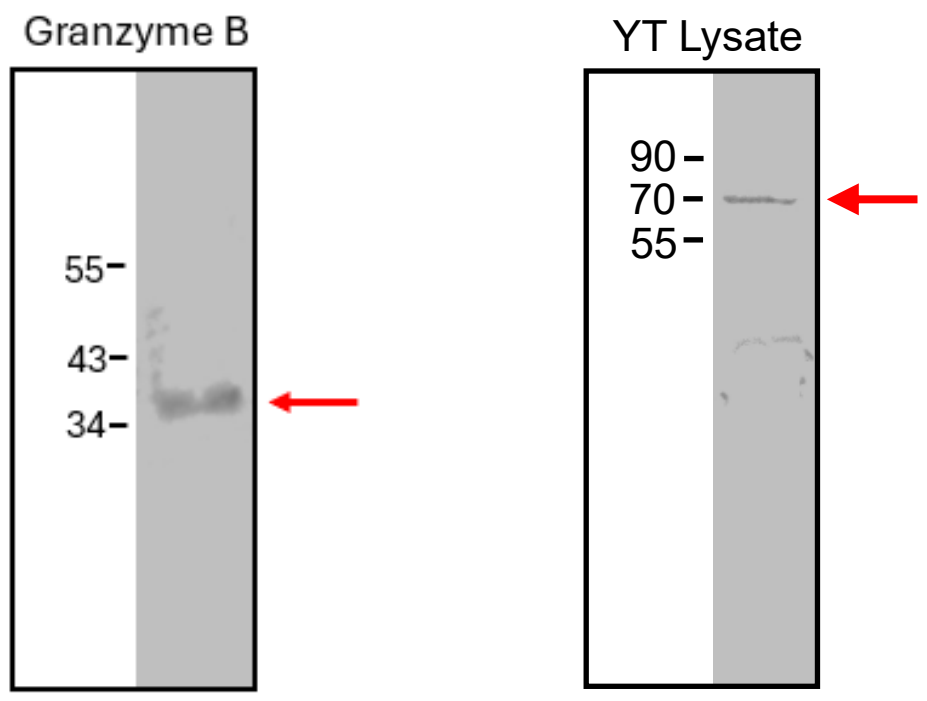

Figure S8. Validation of vitronectin antibody specificity. Western blot demonstrating specificity of the anti-vitronectin primary antibody (Abcam, ab45139). *Left panel:* Recombinant human vitronectin protein was used as a positive control. The antibody detects a distinct, specific band at ~75 kDa (red arrow), which corresponds to the expected molecular weight of the glycosylated protein. *Right panel:* Western blot of retinal pigment epithelium (RPE) cell lysates (3 independent replicates) probed with the same antibody. A distinct band is detected at ~55 kDa (red arrow), corresponding to the expected molecular weight of endogenous vitronectin in these cells.

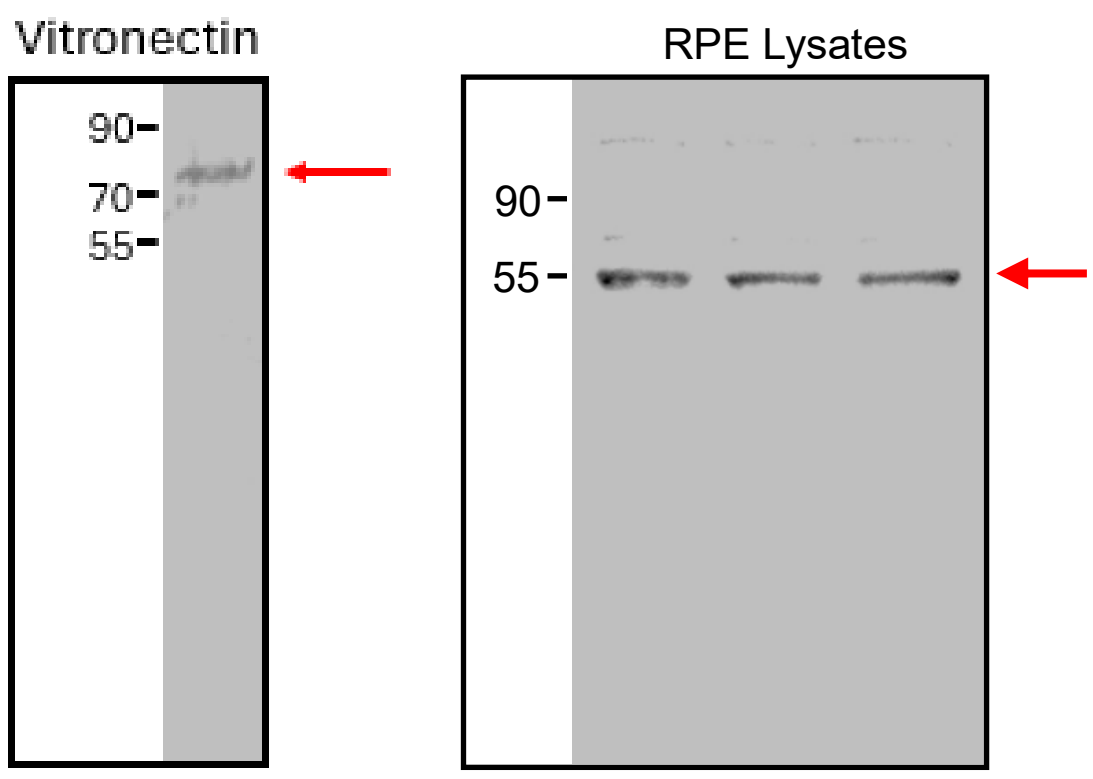

Figure S9. Localization of sub-RPE deposits in high-risk (CC) donor eyes. DAPI-only fluorescence imaging at 20X (*left panel*) and 40X (*right panel*) magnification providing context for the region shown in Figure 2B. White arrows indicate the nuclei of intact RPE cells flanking a large sub-RPE deposit, confirming its sub-RPE localization.

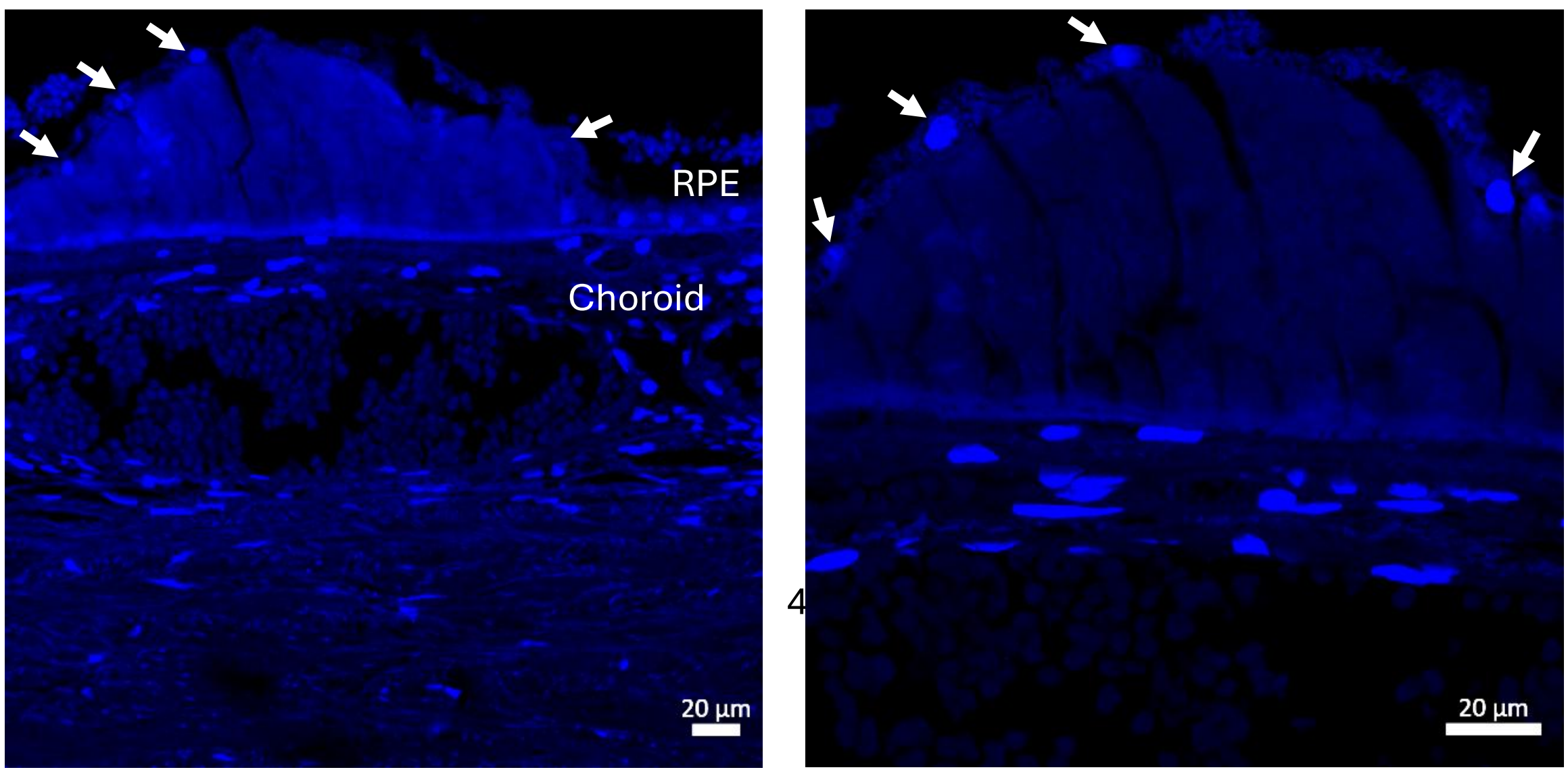

Figure S10. Sub-RPE vitronectin localization in protective (TT) control eyes.

Representative confocal images of a TT donor eye cross-section demonstrating age-related baseline deposit formation. *Left panel*: Vitronectin immunoreactivity (red), showing both RPE/choroid signal and a small sub-RPE deposit (white arrow). *Right panel*: Merged image (vitronectin, red; mast cell tryptase, green; DAPI, blue), showing the localization of the vitronectin-positive deposit beneath the RPE nuclei. Sub-RPE deposits in TT eyes are smaller and fewer in number compared to high-risk CC eyes. Scale bar= 20µm.

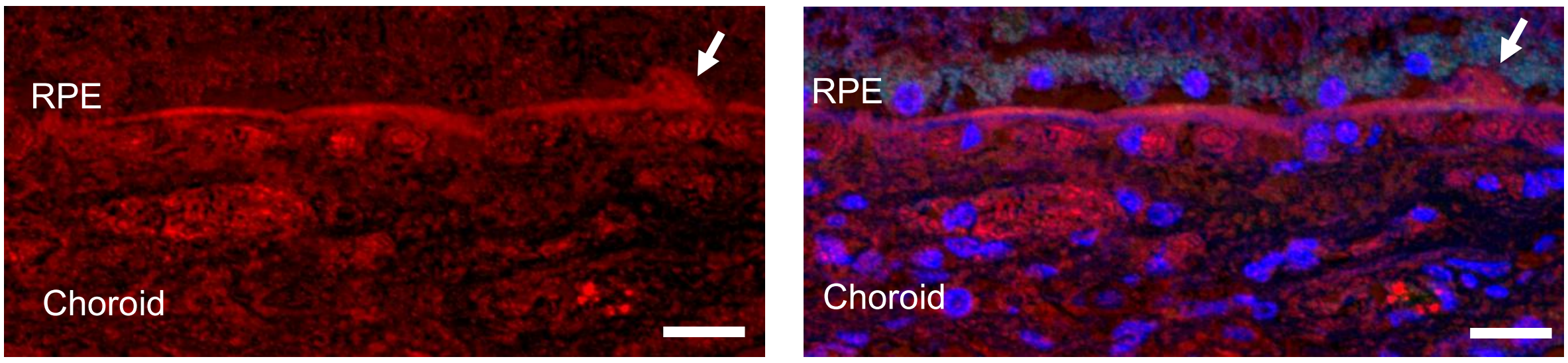

Table S1. Characteristics of post-mortem human donor eyes used in the study.

Sample Characteristics	***TT Genotype n=5***	***CC Genotype n=5***	***P-value***
Age in years, Mean (SEM)	62.6 (4.0)	61.0 (5.3)	*>0.99*
Gender, N (%)			
Male	3 (60%)	4 (80%)	
Female	2 (40%)	0 (0%)	
N/A	0 (0%)	1 (20%)	

Table S2. Age, sex and genotype of AMD and control plasma used in the study.

Sample Characteristics	***Mean (SEM) or N(%)***
Age (years)	75.6 (2.8)
Gender:	
Male	7 (30%)
Female	16 (70%)
CFH Y402H genotype:	
CC	8 (35%)
TT	14 (61%)
CT	1 (4%)

Table S3. RPE-specific gene expression in bulk RNA-seq dataset.

Normalized counts showing stable baseline expression of key RPE markers (*RBP1*, *TJP1 and BEST1*) in both control and Granzyme B-stimulated cultures, consistent with the known phenotype of the immortalized ARPE-19 cell line.

Gene symbol	Gene Name	Control (Avg Normalized Counts, n=3)	GzmB-stimulated (Avg Normalized Counts, n=3)	Log2 Fold Change	Adj. p-value
RBP1	Retinal binding protein 1	945.63	679.08	-0.082	0.884
TJP1	Tight junction protein 1	3676.18	3951.559	0.104	0.708
BEST1	Bestrophin 1	201.45	185.30	-0.119	0.843
